# Perinatal Derivatives: Where Do We Stand? A Roadmap of the Human Placenta and Consensus for Tissue and Cell Nomenclature

**DOI:** 10.3389/fbioe.2020.610544

**Published:** 2020-12-17

**Authors:** Antonietta Rosa Silini, Roberta Di Pietro, Ingrid Lang-Olip, Francesco Alviano, Asmita Banerjee, Mariangela Basile, Veronika Borutinskaite, Günther Eissner, Alexandra Gellhaus, Bernd Giebel, Yong-Can Huang, Aleksandar Janev, Mateja Erdani Kreft, Nadja Kupper, Ana Clara Abadía-Molina, Enrique G. Olivares, Assunta Pandolfi, Andrea Papait, Michela Pozzobon, Carmen Ruiz-Ruiz, Olga Soritau, Sergiu Susman, Dariusz Szukiewicz, Adelheid Weidinger, Susanne Wolbank, Berthold Huppertz, Ornella Parolini

**Affiliations:** ^1^Centro di Ricerca E. Menni, Fondazione Poliambulanza-Istituto Ospedaliero, Brescia, Italy; ^2^Department of Medicine and Ageing Sciences, G. d’Annunzio University of Chieti-Pescara, Chieti, Italy; ^3^StemTeCh Group, G. d’Annunzio Foundation, G. d’Annunzio University of Chieti-Pescara, Chieti, Italy; ^4^Division of Cell Biology, Histology and Embryology, Gottfried Schatz Research Center, Medical University of Graz, Graz, Austria; ^5^Department of Experimental, Diagnostic and Specialty Medicine, Unit of Histology, Embryology and Applied Biology, University of Bologna, Bologna, Italy; ^6^Ludwig Boltzmann Institute for Experimental and Clinical Traumatology, AUVA Research Center, Austrian Cluster for Tissue Regeneration, Vienna, Austria; ^7^Department of Molecular Cell Biology, Institute of Biochemistry, Life Sciences Center, Vilnius University, Vilnius, Lithuania; ^8^Systems Biology Ireland, School of Medicine, University College Dublin, Dublin, Ireland; ^9^Department of Gynecology and Obstetrics, University Hospital Essen, University Duisburg-Essen, Essen, Germany; ^10^Institute for Transfusion Medicine, University Hospital Essen, University of Duisburg-Essen, Essen, Germany; ^11^Shenzhen Engineering Laboratory of Orthopaedic Regenerative Technologies, Department of Spine Surgery, Peking University Shenzhen Hospital, Shenzhen, China; ^12^Institute of Cell Biology, Faculty of Medicine, University of Ljubljana, Ljubljana, Slovenia; ^13^Instituto de Biopatología y Medicina Regenerativa, Centro de Investigación Biomédica, Universidad de Granada, Granada, Spain; ^14^Departamento de Bioquímica y Biología Molecular III e Inmunología, Universidad de Granada, Granada, Spain; ^15^Unidad de Gestión Clínica Laboratorios, Hospital Universitario Clínico San Cecilio, Granada, Spain; ^16^Vascular and Stem Cell Biology, Department of Medical, Oral and Biotechnological Sciences, G. d’Annunzio University of Chieti-Pescara, CAST (Center for Advanced Studies and Technology, ex CeSI-MeT), Chieti, Italy; ^17^Department of Life Science and Public Health, Università Cattolica del Sacro Cuore, Rome, Italy; ^18^Stem Cells and Regenerative Medicine Lab, Department of Women’s and Children’s Health, University of Padova, Fondazione Istituto di Ricerca Pediatrica Città della Speranza, Padua, Italy; ^19^The Oncology Institute “Prof. Dr. Ion Chiricuta”, Cluj-Napoca, Romania; ^20^Department of Morphological Sciences-Histology, Iuliu Haţieganu University of Medicine and Pharmacy, Cluj-Napoca, Romania; ^21^Department of Pathology, IMOGEN Research Center, Cluj-Napoca, Romania; ^22^Department of General and Experimental Pathology with Centre for Preclinical Research and Technology (CEPT), Medical University of Warsaw, Warsaw, Poland; ^23^Fondazione Policlinico Universitario “Agostino Gemelli” IRCCS, Rome, Italy

**Keywords:** perinatal, derivatives, tissues, placenta, fetal annexes, cells, consensus nomenclature

## Abstract

Progress in the understanding of the biology of perinatal tissues has contributed to the breakthrough revelation of the therapeutic effects of perinatal derivatives (PnD), namely birth-associated tissues, cells, and secreted factors. The significant knowledge acquired in the past two decades, along with the increasing interest in perinatal derivatives, fuels an urgent need for the precise identification of PnD and the establishment of updated consensus criteria policies for their characterization. The aim of this review is not to go into detail on preclinical or clinical trials, but rather we address specific issues that are relevant for the definition/characterization of perinatal cells, starting from an understanding of the development of the human placenta, its structure, and the different cell populations that can be isolated from the different perinatal tissues. We describe where the cells are located within the placenta and their cell morphology and phenotype. We also propose nomenclature for the cell populations and derivatives discussed herein. This review is a joint effort from the COST SPRINT Action (CA17116), which broadly aims at approaching consensus for different aspects of PnD research, such as providing inputs for future standards for the processing and *in vitro* characterization and clinical application of PnD.

## Introduction

In the past 20 years, there have been significant advances in the research and understanding of the biology of the placenta and its derivatives. Initially, the placenta drew attention as an interesting cell source due to its early embryological origin suggesting that cells derived from the placenta could possess unique plasticity and differentiation properties (Bailo et al., 2004). In addition, the placenta displays favorable logistical issues, such as the fact that the human term placenta is readily available at the time of delivery.

We now know that perinatal derivatives are promising for a wide range of regenerative medicine applications due to their differentiation capabilities but mainly due to their unique immune modulatory properties. As a matter of fact, many preclinical studies and initial clinical trials have demonstrated that perinatal derivatives may represent important tools for restoring tissue damage or promoting regeneration and repair of the tissue microenvironment (Caruso et al., 2012; Cirman et al., 2014; Jerman et al., 2014; Silini et al., 2015; Joerger-Messerli et al., 2016; Magatti et al., 2016; Couto et al., 2017; Silini et al., 2017; Bollini et al., 2018; Pogozhykh et al., 2018; Ramuta and Kreft, 2018; Verter et al., 2018; Silini et al., 2019; Ramuta et al., 2020). The term “perinatal” refers to birth-associated tissues that are obtained from term placentas and fetal annexes and more specifically refers to the amniotic/amnionic (herein referred to as amniotic due to its prevalence in literature) membrane, chorionic membrane, chorionic villi, umbilical cord (including Wharton’s jelly), the basal plate (including maternal and fetal cells), and the amniotic fluid. The term “derivatives” is used to refer to the cells isolated from placental tissues, and the factors that these cells release, referred to as their secretome or conditioned medium (including free nucleic acids, soluble proteins, lipids, and extracellular vesicles (such as exosomes, microvesicles and apoptotic bodies). Thus, perinatal derivatives (PnD) include different birth-associated tissues, the cells isolated thereof, and the factors secreted by the cells [fractionated (free-floating factors, extracellular vesicles, extracellular matrix components including proteins, glycosaminoglycans, and glycoconjugates) and unfractionated conditioned medium].

Over a decade ago, in 2008, the consensus from the *First International Workshop on Placenta-Derived Stem Cells* was published (Parolini et al., 2008). The consensus focused on cells isolated from the amniotic and chorionic parts of the fetal membranes and established the minimal criteria for the definition of mesenchymal stromal cells (MSC) derived from these membranes. In accordance to the criteria established for other MSC sources (Dominici et al., 2006), the criteria established at the *First International Workshop on Placenta-Derived Stem Cells* focused on adherence to plastic, formation of fibroblast-like colony-forming units, differentiation potential toward one or more lineages, including osteogenic, adipogenic, or chondrogenic lineages, and specific cell surface antigen expression from *in vitro* passages 2 to 4 (Parolini et al., 2008). In addition, the criteria included one other specific aspect, the determination of the fetal or maternal origin of the perinatal cells (Parolini et al., 2008).

During the last two decades, the literature published on perinatal derivatives has grown exponentially. Specific cells such as MSC have been isolated and characterized from different perinatal tissues, such as the fetal membranes (In ’t Anker et al., 2004; Soncini et al., 2007; Wolbank et al., 2010), chorionic villi (Fukuchi et al., 2004; Igura et al., 2004; Portmann-Lanz et al., 2006; Castrechini et al., 2010), decidua (In ’t Anker et al., 2004; Araújo et al., 2018; Ringden et al., 2018; Guan et al., 2019), and umbilical cord (Wang et al., 2004b; Troyer and Weiss, 2008; La Rocca et al., 2009; Hartmann et al., 2010).

The significant increase in acquired knowledge has been paralleled with the evident need for the establishment of updated criteria and consensus policies for the characterization of PnD. Thus, this review aims at providing an updated and extended consensus starting from the policies published in 2008, which were specifically related to cells from fetal membranes (Parolini et al., 2008), and at addressing specific issues related to the proper and transparent definition of PnD, relating not only to fetal membranes but also to all other regions and perinatal tissues.

**FIGURE 1:**
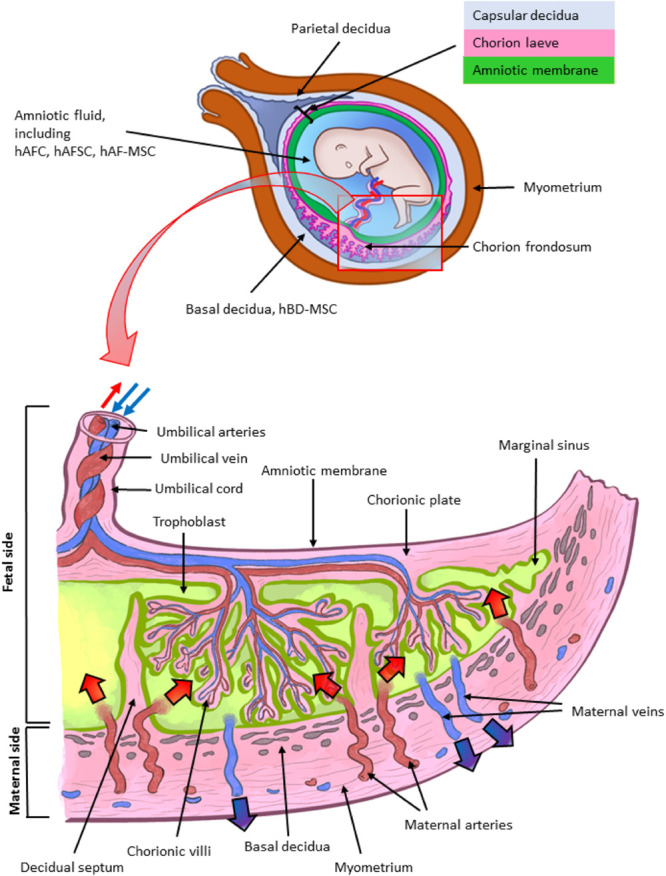
Architecture of the human term placenta. General overview of the relationship between the basal decidua (maternal side/component of the human placenta) and the fetal side/component of the human placenta represented by the chorion frondosum, the chorionic plate and the fused amniotic membrane (placental portion). The residual portion of the amniotic membrane (reflected portion) adheres to the chorion laeve (so called because it is devoid of villi) which is in touch with the capsular decidua. The amniotic membrane surrounds the amniotic cavity containing amniotic fluid with different types of detached cells. The magnified scheme shows the different parts of the term placental architecture. hBD-MSC, human basal decidua-mesenchymal stromal cells; hAFC, human amniotic fluid cells; hAFSC, human amniotic fluid stem cells; hAF-MSC, human amniotic fluid-mesenchymal stromal cells.

One issue that must be addressed is related to defining PnD. In its simplest form, this means establishing a reference nomenclature for each derivative that can be isolated from all perinatal, birth-associated tissues. Birth associated or perinatal tissues and organs, such as the human placenta, are complex and are comprised of different tissues (as mentioned above, amniotic membrane, chorionic membrane, chorionic villi, umbilical cord, basal plate including fetal trophoblast cells and maternal uterine cells, and amniotic fluid) (Figure 1). Even today, there is much confusion regarding the identification and location of the specific perinatal tissues and cells. In the current literature the nomenclature used does not necessarily highlight the true differences between cells. At the same time, not all cells can simply be referred to as “placenta-derived stem cells” (Oliveira and Barreto-Filho, 2015), without taking into consideration the exact tissue from which they were derived. A proper and clearly defined nomenclature is absolutely necessary to understand which cells are isolated and used in cell cultures. Incorrect nomenclature and definition of cells ultimately impact the correct identification of the cells and/or derivatives obtained and hinder the direct comparison of results among different research groups.

Reference nomenclature should be established followed by a clear indication of the precise localization of cells in perinatal tissues. This should be followed by the next crucial step, the definition of the phenotype of cells and more specifically the markers that will serve as reference standards to identify specific cell types. To this regard, it is important to consider an aspect specifically related to perinatal cells and that is the determination of the fetal or maternal origin (Parolini et al., 2008). This is critical since the detection of maternal cells may depend on cell expansion in *in vitro* culture. For example, we previously demonstrated that genomic Polymerase Chain Reaction amplification was not able to determine the presence of maternal cells in the freshly isolated mesenchymal fractions of both the amnion and chorion (Soncini et al., 2007). However, after several cell passages in culture, maternal cells were detected in cell populations from the chorionic membrane, while those from the amniotic membrane did not show the presence of maternal alleles (Soncini et al., 2007). Hence, this may well result in working with a mixture of maternal and fetal cells, while the intent was to just work with fetal cells.

Another important aspect relates to cell culture expansion, because specific characteristics of the cells change, including phenotype and expression of specific proteins. This may result in the impression that one may be working with different cells compared to those isolated from perinatal tissues. In the case of human amniotic membrane MSC (hAMSC) and human amniotic membrane epithelial cells (hAEC) from placenta, it has previously been demonstrated that cell culture up to passage 4 after isolation (passage 0) can induce changes in the expression of cell markers. Such changes include the significant increase in adhesion molecules (e.g., CD49b, CD49d) and the significant decrease of CD14, CD45, and HLA-DR expression on hAMSC, as well as the significant increase of CD13, CD44, CD105, CD146 expression on cultures of hAEC (Stadler et al., 2008; Magatti et al., 2015).

Here, considering the numerous publications and the increasing interest in perinatal derivatives, we address specific issues that are relevant for the clear and precise definition/characterization of perinatal cells, starting from an understanding of the development of the human placenta, its structure, and the different cell populations that can be isolated from the different perinatal tissues. In addition, we describe where the cells are located within the placenta and provide an atlas of the human placenta. We also describe cell morphology and phenotype and propose nomenclature for the cell populations and derivatives discussed herein. The proposed nomenclature will be crucial to lay the foundation for the consistency in the scientific community when referring to PnD. This review is a joint effort from the COST SPRINT Action (CA17116), which broadly aims at approaching consensus for different aspects of PnD research, such as providing inputs for future standards for the processing, *in vitro* characterization and clinical application of PnD.

## Development of the Human Placenta

The placenta is the first organ to develop in mammals. It is essential for the successful growth of the embryo, and later the fetus, and its crosstalk with the uterine maternal compartment is indispensable. Its fundamental role is underlined by the fact that impaired formation of placental tissues leads to pregnancy disorders, such as preeclampsia, fetal growth restriction, recurrent miscarriage, and stillbirth. The embryo-maternal interface is based on an intimate and controlled relationship between the conceptus and the mother. The placenta and extraembryonic membranes maintain this essential contact for supporting the development of the new organism by acquiring oxygen and nutrients, eliminating waste, and avoiding immune rejection. However, despite its importance in reproductive outcome, there is still a limited understanding about human placental development, mainly due to ethical and logistic difficulties in investigating it in the early stages, as well as in extrapolating data from other species (Enders and Carter, 2004; Turco and Moffett, 2019).

**FIGURE 2:**
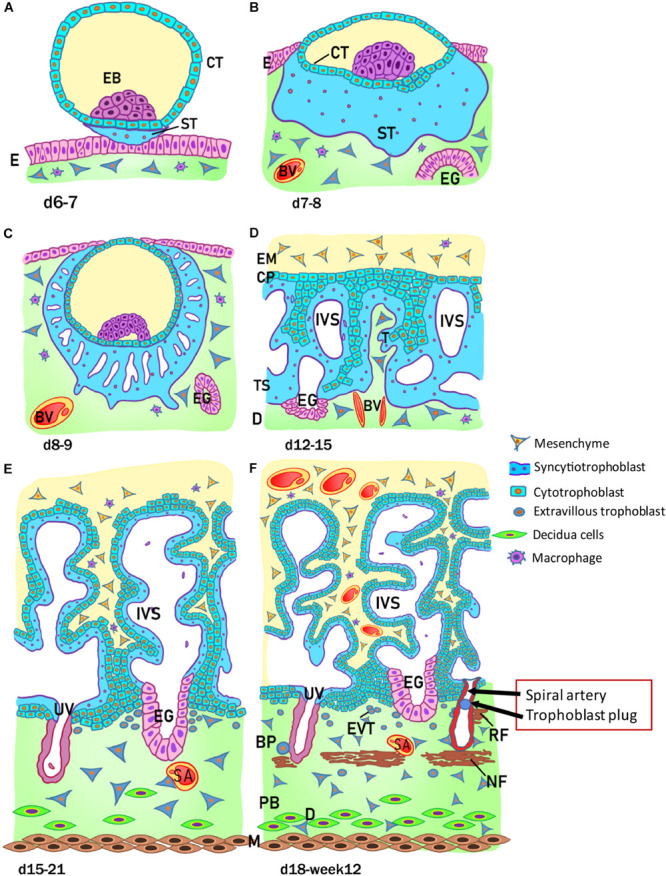
Stages of placental development. **(A)** Implantation at 6 to 7 days (d) after conception; **(B)** prelacunar period (7 to 8 days); **(C)** beginning of lacunar period (8 to 9 days); **(D)** transition from lacunar period to primary villus stage (12 to 15 days); **(E)** secondary villus stage (15 to 21 days); and **(F)** tertiary villus stage (18 days to week 12). BP, basal plate; BV, blood vessel; CP, primary chorionic plate; CT, cytotrophoblast; D, decidua; E, endometrial epithelium; EB, embryoblast; EG, endometrial gland; EM, extraembryonic mesoderm; EVT, extravillous trophoblast; IVS, intervillous space; M, myometrium; NF, Nitabuch fibrinoid; PB, placental bed; RF, Rohr fibrinoid; SA, spinal artery; ST, syncytiotrophoblast; T, trabeculae; TS, trophoblastic shell; UV, umbilical vein. (Redrawn and modified from Kaufmann, 1981).

The development of the human placenta begins from the first days after conception (Figure 2). Fertilization takes place in the ampulla, the third portion of the uterine tube, and consists of the fusion of the female gamete, the egg, with the male gamete, the sperm. After the formation of the zygote at fertilization, the subsequent cleavage phase leads to a series of mitotic divisions which give rise to the formation of a compact mass, the morula (early and late stage), and subsequently to the blastocyst consisting of a single layered epithelial cover, called trophoblast or trophectoderm, which surrounds and encloses the cavity of the blastocyst (blastocoel). Inside this cavity there is a group of concentrated and polarized cells that constitute the inner cell mass (ICM), the embryoblast (Figure 2A). Following blastocyst formation implantation takes place in the uterine wall 6-7 days after fertilization. At the time of blastocyst attachment, trophoblast/trophectoderm cells in direct contact with the endometrial epithelium start to fuse and generate the first trophoblast cell type, the syncytiotrophoblast. Apparently, only this multinucleated structure is able to penetrate through the endometrial epithelium. The syncytiotrophoblast and the underlying layers of mononucleated cytotrophoblasts still surround the ICM, which is committed to create all embryonic tissues, the umbilical cord and the epithelium of the amniotic membrane. Following implantation, the ICM gets surrounded by a ball-shaped placenta (Figure 2B). This is the prelacunar phase of placental development (Benirschke et al., 2006).

As shown in Figure 2, at this early stage of development (day 6-7) the trophoblast/trophectoderm does not directly contribute to the development of the embryo but constitutes the fetal portion of the placenta: the chorion. In this phase, the cells of the ICM differentiate into two layers: the hypoblast, or primitive endoderm, a layer of small cubic cells facing the blastocoel cavity, and the epiblast, or primitive ectoderm, a layer of cylindrical cells facing the embryonic pole and adjacent to what will become the amniotic cavity. Together these two cell layers form the bilaminar embryonic disk. At the 8^*th*^ day post conception, some cells from the epiblast migrate and position between the cytotrophoblast and the underlying embryonic disk, creating a small space that will later become the amniotic cavity. The cells derived from the epiblast, which surround the future amniotic cavity, are called amnioblasts and will give rise to the amniotic epithelium. Subsequently, the cytotrophoblast secretes a spongy layer of acellular material, called the extraembryonic reticulum, which will give rise to extraembryonic mesoderm after invasion of the migratory cells from the epiblast. This layer surrounds the yolk sac and the amniotic cavity and, subsequently, will constitute the amniotic and chorionic mesoderm (extraembryonic somatopleuric mesoderm). At this point fluid-filled spaces (lacunae) begin to develop within the syncytial mass that enlarge and merge leading to the formation of a lacunar system (lacunar stage) (Figure 2C). As a consequence of the erosion of the endothelial lining of the maternal capillaries, lacunae are filled with maternal blood giving rise to a primitive filling of the lacunae with maternal blood, while a utero-placental circulation will only develop later in pregnancy. At day 13 post conception (Figure 2D) the cytotrophoblasts proliferate locally and penetrate the syncytiotrophoblast forming columns of cells surrounded by the syncytium, the primary villi. Subsequently, cells of the extraembryonic somatopleuric mesoderm penetrate inside the primary villi and grow in the direction of the decidua, i.e. the maternal component of the placenta, to form the secondary villi (Figure 2E). From the end of the third week (Figure 2F), the mesodermal cells inside the villi begin to differentiate into endothelial and blood cells and thus form small blood vessels that give rise to the capillary system of the villi and to the tertiary or definitive villi. The villous cytotrophoblast progressively penetrates the syncytiotrophoblast until they reach the endometrium to form trophoblastic cell columns and the external cytotrophoblastic shell. At the point where the cytotrophoblast is in touch with the maternal decidua, single cytotrophoblast cells leave the shell to invade into the decidua as extravillous trophoblast (EVT) in a process closely resembling epithelial-mesenchymal transition (EMT). During the second month of pregnancy, due to the increased volume of the amnion, the amniotic membrane fuses with the chorion leading to the formation of the amnio-chorionic membrane. Within the first trimester of pregnancy the organization and structure of the placenta is established.

## Structure of the Early Placenta

**FIGURE 3:**
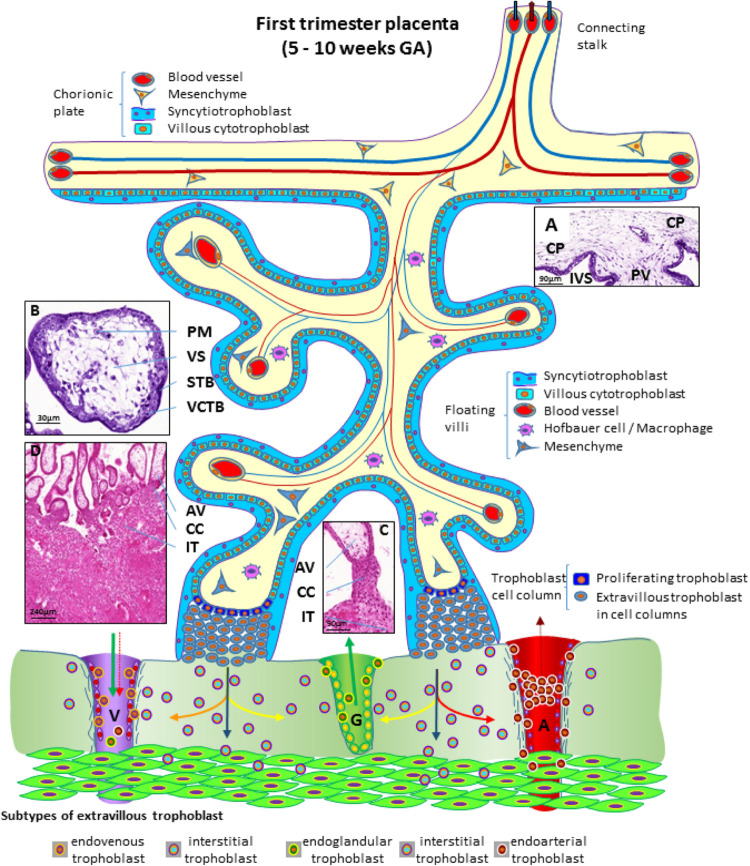
Schematic representation of a human placenta during the first trimester of pregnancy. The chorionic plate represents the embryonic side of the placenta from which placental villi grow into the intervillous space. Anchoring villi are connected to the uterine wall by trophoblast cell columns from which extravillous trophoblasts invade into uterine tissues. From these sites interstitial trophoblast invades into the uterine stroma, differentiating into endoglandular trophoblast invading uterine glands, endovenous trophoblast, invading uterine veins and endoarterial trophoblast invading into uterine spiral arteries Histological images of **(A)** first trimester chorionic plate with a placental villus extending into the intervillous space, **(B)** first trimester mesenchymal villus with the cover of villous trophoblast and the mesenchymal villous stroma, **(C)** anchoring villus that is attached to the uterine wall by a trophoblast cell column, **(D)** first trimester placenta showing a number of anchoring villi attached to the uterine wall by trophoblast cell columns. Within the uterine wall a huge amount of interstitial trophoblast invades towards vessels, glands and the myometrium. A, uterine spiral artery; AV, anchoring villus; CC, trophoblast cell column; CP, chorionic plate; G, uterine gland; GA, gestational age; IT, interstitial trophoblast; IVS, intervillous space; PM, placental macrophage (Hofbauer cell); PV, placental villus; STB, syncytiotrophoblast; V, uterine vein; VCTB, villous cytotrophoblast; VS, villous stroma.

During the first trimester of pregnancy, the placenta develops all the structures needed for a sufficient supply of nutrients to the embryo and, subsequently, the fetus during pregnancy (Huppertz et al., 2014). Several structures can be identified in a first trimester placenta (from embryo to mother) (Figure 3), at which time, the amnion is not fully developed, hence a specific layer of amniotic membrane cannot be found covering the chorionic plate at this stage of pregnancy. The first layer of a first trimester placenta from the embryo’s perspective is the chorionic mesenchyme, a vascularized connective tissue where the connecting vessels between placental villi and connecting stalk (that later develops into the umbilical cord) can be found. As placental villi grow from the side of the chorionic layer facing the intervillous space, it is referred to as chorionic plate of the placenta (Figure 3A). At the side towards the intervillous space, the chorionic plate is still covered with the same layers as the placental villi, syncytiotrophoblast and villous cytotrophoblast (Figure 3A).

From the chorionic plate, larger villi grow into the intervillous space (Figure 3A) and form tree-like structures, the villous trees. Longitudinal growth in combination with branching and sprouting generates these tree-like structures (Figure 3). During the first trimester of pregnancy, until week 12 to week 14, there is no flow of maternal blood through the intervillous space surrounding the placental villi.

The placental villi are covered by the syncytiotrophoblast that comes in direct contact with maternal plasma/blood. Directly underneath the syncytiotrophoblast, a complete layer of villous cytotrophoblast can be found (Figure 3B). These cells represent the proliferating progenitor cells of this epithelial layer. Some of the progenitors’ sibling cells differentiate and fuse with the overlying syncytiotrophoblast. Connective tissue derived from the chorionic mesenchyme fills the cores of the placental villi. Within this tissue, blood vessels, blood cells as well as placental macrophages (Hofbauer cells) (Figure 3B) develop prior to the connection to the embryonic blood system via the connecting stalk (Huppertz and Peeters, 2005; Demir et al., 2007).

During the first half of the first trimester of pregnancy, the freely floating villi are very primitive and are classified as mesenchymal villi. At about 8 to 10 weeks of pregnancy, the first mesenchymal villi differentiate into immature intermediate villi, characterized by stromal channels where placental macrophages can easily be visualized. The placental villi that connect to the uterine wall are called anchoring villi (Figure 3C). This is the site where the placenta is anchored to the uterine wall and where trophoblastic cell columns (Figure 3C) are formed as sources for all extravillous trophoblast cells invading into the decidua and the inner third of the myometrium of the placental bed (interstitial trophoblast) (Figure 3D). From the interstitial trophoblast (Figures 3C,D) a variety of other subtypes of extravillous trophoblast develop to allow proper nutritional support of the embryo and fetus throughout pregnancy. The trophoblast invades into the uterine spiral arteries (endoarterial trophoblast), the uterine veins (endovenous trophoblast), the uterine glands (endoglandular trophoblast), and finally into the uterine lymph vessels (endolymphatic trophoblast) (Figure 3) (Moser et al., 2018b). The endoarterial trophoblast blocks the flow of maternal blood into the intervillous space and only blood plasma is able to pass through these trophoblast plugs (Figure 3: dashed red arrow in the invaded artery) and reaches the placenta. Additionally, due to invasion by endoglandular trophoblast, secretion products of uterine glands flow into the intervillous space as well (Figure 3: green arrow in the invaded gland). All of this is transferred back into the maternal system by utero-placental veins (Figure 3: green arrow and dashed red arrow in invaded vein), connected to the placenta by invasion of endovenous trophoblast (Figure 3) (Moser et al., 2018b; Huppertz, 2019).

## Structure of the Term Placenta

**FIGURE 4:**
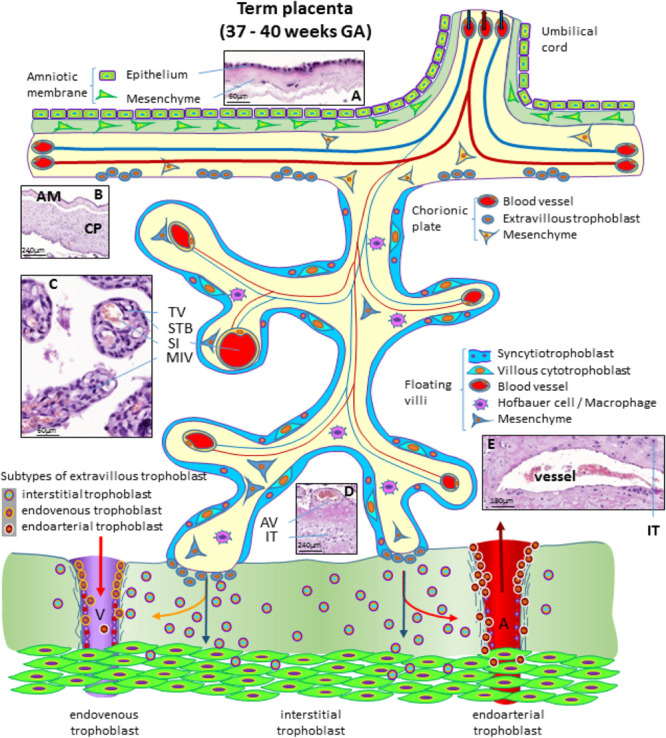
Schematic representation of a human placenta at term. The amniotic membrane is the layer closest to the fetus and is attached to the chorionic plate mesenchyme from which large stem villi reach into the intervillous space. The villous trees are fully differentiated and have a large number of terminal villi where enlarged capillaries, sinusoids, allow a higher exchange rate between maternal and fetal blood. Anchoring villi are still connected to the uterine wall, while trophoblast cell columns are exhausted. Spiral arteries invaded by endoarterial trophoblast and uterine veins invaded by endovenous trophoblast can still be found in the placental bed allowing the constant flow of maternal blood into the placenta and the drainage back into the maternal circulation (red arrows in artery and vein). Histological images of **(A)** term amniotic membrane with epithelium and avascular mesenchyme, **(B)** term chorionic plate covered by the amniotic membrane, **(C)** placental villi of a term placenta with a sinusoid in a terminal villus and a neighboring mature intermediate villus, **(D)** anchoring villus that is attached to the uterine wall where interstitial trophoblast can be found, **(E)** vessel in the basal plate of a term placenta. The vessel is surrounded by interstitial trophoblast. A, uterine spiral artery; AM, amniotic membrane; AV, anchoring villus; CP, chorionic plate; GA, gestational age; IT, interstitial trophoblast; MIV, mature intermediate villus; SI, sinusoid; STB, syncytiotrophoblast; TV, terminal villus; V, uterine vein.

At the end of pregnancy, the placenta has all the structures that were needed to supply the fetus with sufficient amounts of nutrients and gases, as well as to allow excretion of waste products (Huppertz, 2008). In the term placenta, the following structures are present (from fetus to mother) as shown in Figure 4.

The fetus bathes in the amniotic fluid. The outer border of the fluid-filled cavity is demarcated by the amniotic epithelium, a cuboidal and mostly single layered epithelium (Figures 4A,B). Under the basement membrane, the amniotic mesenchyme can be found, an avascular connective tissue. Amniotic epithelium and mesenchyme form the amnion, which surrounds the embryo (Figure 4B).

The next layer is the chorionic mesenchyme. This connective tissue is vascularized and contains the vessels between placental villi and umbilical cord. At the site of the decidua basalis, this layer is called chorionic plate (Figure 4B), while at the site of the fetal membranes it is referred to as chorionic layer of the fetal membranes, the chorion laeve. At the surface of the chorionic plate towards the intervillous space of the placenta, some remnants of extravillous trophoblast may be found (Figure 4). From the chorionic plate, large stem villi reach into the intervillous space and are the trunks of the tree-like structures, the villous trees giving rise to the chorion frondosum. In a term placenta, differentiation of the villous structure leads to a completely different set of villi compared to the first trimester of pregnancy. At term, most of the villi (40%) are terminal villi that are the site of direct transfer of nutrients and gasses between maternal and fetal blood (Figure 4C). Terminal villi can be seen as leaves of a tree, while the underlying mature intermediate villi (Figure 4C) are the connecting branches of the tree, making up about 25% of the total villous volume at term. The placental villi are covered by the syncytiotrophoblast that comes in direct contact to maternal blood and releases huge amounts of fetal material into the maternal circulation (Huppertz et al., 1998). Due to the huge expansion of the villous stroma, the syncytiotrophoblast at term is much thinner than in the first trimester. Also, the layer of villous cytotrophoblast has become discontinuous and only occasionally, single villous cytotrophoblast cells can be identified (Figure 4). The villous stroma is fully differentiated with large caliber arteries and veins in stem villi and sinusoids, enlarged capillaries, in the terminal villi (Figure 4C). Placental macrophages, Hofbauer cells, are present in each and every villus of the placenta.

At term, the anchoring villi are still attached to the uterine wall (Figure 4D). However, the trophoblast cell columns are exhausted and no longer present as real columns (Figure 4D). Interstitial trophoblast can be found in the basal plate of the placenta (Figure 4D) as well as in the placental bed surrounding luminal structures like arteries and veins (Figure 4E).

At the end of pregnancy, trophoblast that has invaded into uterine spiral arteries (endoarterial trophoblast) and utero-placental veins (endovenous trophoblast) can still be found. Invaded endometrial glands are hardly visible at term. In addition, at term the endoarterial trophoblast has led to a widening of uterine spiral arteries to allow a constant flow of maternal blood into the intervillous space (Figure 4: red arrow in the invaded artery) (Huppertz, 2019). Maternal blood is drained back into the maternal circulation by utero-placental veins (Figure 4: red arrow in invaded vein), connected to the placenta by invasion of endovenous trophoblast (Figure 4).

## Cells Isolated From the Term Placenta and Fetal Annexes

### Human Placenta Cells (hPC)

Several cell types can be obtained and expanded from the different regions of the human placenta and the fetal annexes. Human placenta cells (hPC) is a generic term used to refer to any type of cell that can be isolated from term placenta; the most prominent being epithelial cells, mesenchymal stromal cells (MSC), endothelial, and hematopoietic cells.

Amongst these, human placenta MSC (hPMSC) is a general term commonly used to refer to MSC from various perinatal tissues. hPMSC from the different tissues described herein possess similar characteristics in accordance with the minimal consensus criteria reported for MSC from other adult tissues, such as bone marrow (Dominici et al., 2006) and established also for placenta-derived cells during the *First International Workshop on Placenta-Derived Stem Cells* held in Brescia, Italy in 2007. This includes the expression of CD90, CD73, and CD105, and the lack of expression of CD45, CD34, CD14, and HLA-DR (Parolini et al., 2008).

In order to advise the scientific community on the precise localization and nomenclature of perinatal tissues and cells, in the following paragraphs the main characteristics of the different placental/perinatal regions/tissues, and features of their cells will be described. To discriminate similar cells of different origins more accurately a nomenclature will be proposed (Table 1). We also provide representative figures for several cell populations in order to illustrate their localization within given placental sites.

**TABLE 1:** Proposed consensus nomenclature for human perinatal tissues and cells.

Name	Consensus Abbreviation
human placenta cells	hPC
human placenta mesenchymal stromal cells	hPMSC
**human amnio-chorionic membrane**	**hACM**
human amnio-chorionic membrane cells	hACMC
**human amniotic membrane**	**hAM**
human amniotic membrane cells	hAMC
human amniotic membrane epithelial cells	hAEC
human amniotic membrane mesenchymal stromal cells	hAMSC
**human placental amniotic membrane**	**hPAM**
human placental amniotic membrane cells	hPAMC
human placental amniotic membrane epithelial cells	hPAEC
human placental amniotic membrane mesenchymal stromal cells	hPAMSC
**human reflected amniotic membrane**	**hRAM**
human reflected amniotic membrane cells	hRAMC
human reflected amniotic membrane epithelial cells	hRAEC
human reflected amniotic membrane mesenchymal stromal cells	hRAMSC
**human chorion (chorionic membrane)**	**hCM**
human chorionic membrane cells	hCMC
human chorionic mesenchymal stromal cells	hCMSC
**human trophoblast**	**hTB**
human trophectoderm	hTED
human syncytiotrophoblast	hSTB
human cytotrophoblast	hCTB
human villous cytotrophoblast	hVCTB
human extravillous trophoblast	hEVT
human proximal cell column trophoblast	hpCCT
human distal cell column trophoblast	hdCCT
human interstitial extravillous trophoblast	hiEVT
human endovascular extravillous trophoblast	hvasEVT
human endoarterial extravillous trophoblast	hartEVT
human endovenous extravillous trophoblast	hvenEVT
human endoglandular extravillous trophoblast	hglaEVT
human endolymphatic extravillous trophoblast	hlymEVT
human trophoblast stem cells	hTSC
human trophoblast progenitor cells	hTPC
**human chorionic plate**	**hCP**
human chorionic plate cells	hCPC
human chorionic plate mesenchymal stromal cells	hCP-MSC
human chorionic plate mesenchymal stromal cells derived from blood vessels	hCP-MSC-bv
human chorionic plate extravillous trophoblast	hCP-EVT
**human chorionic villi (chorion frondosum)**	**hCV**
human chorionic villi cells	hCVC
human chorionic villi mesenchymal stromal cells	hCV-MSC
human chorionic villi trophoblast cells	hCV-TC
**human chorion laeve**	**hCL**
human chorion laeve mesenchymal stromal cells	hCL-MSC
human chorion laeve extravillous trophoblast	hCL-EVT
**human umbilical cord**	**hUC**
human umbilical cord cells	hUCC
human umbilical cord mesenchymal stromal cells	hUC-MSC
**human umbilical cord amniotic membrane**	**hUC-AM**
human umbilical cord amniotic membrane cells	hUC-AMC
human umbilical cord amniotic epithelial cells	hUC-AEC
human umbilical cord amniotic mesenchymal stromal cells	hUC-AMSC
**human umbilical cord Wharton’s jelly**	**hUC-WJ**
human umbilical cord Wharton’s jelly cells	hUC-WJC
human umbilical cord Wharton’s jelly mesenchymal stromal cells	hUC-WJ-MSC
human umbilical cord sub-amnion Wharton’s jelly mesenchymal stromal cells	hUC-saWJ-MSC
human umbilical cord intermediate Wharton’s jelly mesenchymal stromal cells	hUC-iWJ-MSC
**human umbilical cord vascular region**	**hUC-V**
human umbilical cord perivascular cells	hUC-PVC
human umbilical cord vascular smooth muscle cells	hUC-VSMC
human umbilical cord myofibroblasts	hUC-MF
human umbilical vein endothelial cells	HUVEC
human umbilical artery endothelial cells	HUAEC
human placental endothelial cells	hP-EC
human chorionic villi endothelial cells	hCV-EC
human placenta microvascular endothelial cells	hP-mV-EC
human chorionic plate endothelial cells	hCP-EC
human placental venous endothelial cells (derived from chorionic veins)	hPV-EC
human placental arterial endothelial cells (derived from chorionic arteries)	hPA-EC
**human amniotic fluid**	**hAF**
human amniotic fluid cells	hAFC
human amniotic fluid stem cells	hAFSC
human amniotic fluid mesenchymal stromal cells	hAF-MSC
**human decidua**	**hD**
human decidual stromal cells	hDSC
human decidua predecidual stromal cells	hD-preDSC
human decidua decidualized stromal cells	hD-dDSC
precursors of decidualized stromal cells	preDSC
human decidua mesenchymal stromal cells	hDMSC
**human basal decidua**	**hBD**
human basal decidua predecidual stromal cells	hBD-preDSC
human basal decidua decidualized stromal cells	hBD-dDSC
human basal decidua mesenchymal stromal cells	hBD-MSC
**human parietal decidua**	**hPD**
human parietal decidua predecidual stromal cells	hPD-preDSC
human parietal decidua decidualized stromal cells	hPD-dDSC
human parietal decidua mesenchymal stromal cells	hPD-MSC
**human capsular decidua**	**hCD**
human capsular decidua predecidual stromal cells	hCD-preDSC

*Bold text is used to identify perinatal tissues and to distinguish them from cell populations. See text for more details on the cell types.*

### Human Amnio-Chorionic Membrane (hACM) and Human Amniotic Membrane (hAM)

**FIGURE 5:**
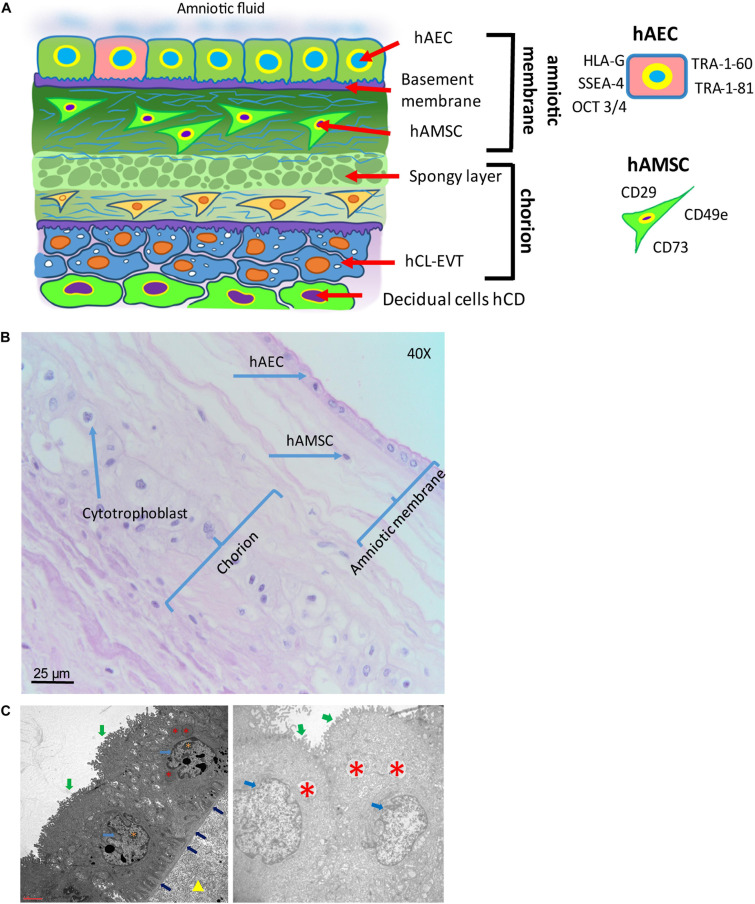
Structure of the human amniotic membrane. **(A)** Schematic representation of the structure of the human amniotic membrane and tissues underneath (chorion laeve and capsular decidua). Human amniotic membrane epithelial cells (hAEC) form, for the most part, a monolayer facing the amniotic fluid. The basement membrane underneath separates the epithelial layer from the avascular amniotic mesoderm including the compact layer devoid of cells in touch with the basement membrane and the fibroblast layer below it containing human amniotic membrane mesenchymal stromal cells (hAMSC). Between amniotic membrane and the vascular chorionic mesoderm there are slender, fluid filled clefts forming an intermediate spongy layer. A basement membrane separates chorionic mesoderm from extravillous trophoblast (hCL-EVT) (embedded in self-secreted matrix-type fibrinoid) which is in touch with maternal capsular decidua (hCD). **(B)** Histological image of human amniotic membrane stained with haematoxylin-eosin staining solution. Magnification: 40x. **(C)** Transmission electron microscopy image showing the ultrastructure of hAEC belonging to the central region of hAM (left panel) or the peripheral region of hAM. Green arrows point at surface microvilli; pale blue arrows point at nuclei; blue arrows point at the basement membrane below hAEC; red asterisks point at different types of granules; orange asterisks point at nucleoli; yellow triangle points at extracellular matrix of the compact layer. Magnification: 3000x.

The human amniotic membrane (hAM) represents the wall of an embryo/fetal annex called the amnion or amniotic sac, which encloses the amniotic cavity and contains the amniotic fluid (AF), (Figure 5). During embryonic development, the enlargement of the amniotic cavity causes the hAM to come in contact with the chorion leading to the formation of the amnio-chorionic membrane (hACM), which is the membrane of the human placenta directly facing the embryo/fetus. It is the general term for the combination of the fetal part of the fetal membranes plus the chorionic plate. Cells isolated from this membrane can be generally referred to as human amnio-chorionic membrane cells (hACMC). The phenotype of hACMC depends on the specific cell type (i.e. epithelial, mesenchymal) and is consistent with those described below.

The hAM is a monolayer of cuboidal-shaped human amniotic membrane epithelial cells (hAEC) with microvilli on the apical surface in direct contact with the AF (Figure 5). An *in situ* investigation revealed the morphological heterogeneity of the different cell populations belonging to different regions identified within the hAM (Centurione et al., 2018). This heterogeneity could be due to the fact that the majority of research laboratories use the hAM that adheres to the chorion laeve (i.e. human reflected amniotic membrane, hRAM), rather than hAM that adheres to the chorionic plate (i.e. human placental amniotic membrane, hPAM). As a matter of fact, Centurione et al., identified four areas of the hAM: the central, intermediate, peripheral, and reflected regions in relation to the umbilical cord (Centurione et al., 2018). Interestingly, the epithelial layer was found to be multilayered in all areas except in the intermediate area, while in the central area the nuclei were located in a higher position compared to the other regions. Furthermore, apoptotic cells were predominantly found in the central area, although, in other areas, budding and detaching cells were present as well.

#### Human Amniotic Membrane Cells (hAMC)

Human amniotic membrane cells (hAMC) are of fetal origin. The structure of the hAM defines a layer of hAEC in direct contact with the AF and a population of amniotic membrane mesenchymal stromal cells (hAMSC) embedded in the underlying connective tissue (Figure 5). In addition, the location of the hAM relative to the chorionic regions (chorionic plate and chorion laeve) determines at least two populations of hAMC, human reflected amniotic membrane cells (hRAMC) and human placental amniotic membrane cells (hPAMC).

Amniotic cells can be isolated from the hAM either as a heterogenous total population or by separate enzymatic digest to obtain hAEC and hAMSC. In most protocols trypsin is used for isolation of hAEC and collagenase for hAMSC (Casey and MacDonald, 1997; Whittle et al., 2000; In ’t Anker et al., 2004; Sakuragawa et al., 2004; Miki and Strom, 2006; Portmann-Lanz et al., 2006; Soncini et al., 2007; Sudo et al., 2007; Tamagawa et al., 2007; Wolbank et al., 2007; Bilic et al., 2008; Magatti et al., 2008; Parolini et al., 2008; Wei et al., 2009; Kang et al., 2012; Lindenmair et al., 2012; Lisi et al., 2012). However, other digestive enzymes including dispase (Soncini et al., 2007; Tamagawa et al., 2007; Bilic et al., 2008; Díaz-Prado et al., 2010) or enzyme combinations (e.g., collagenase/DNase) (Alviano et al., 2007; Soncini et al., 2007; Konig et al., 2012) have also been utilized.

The quality of amniotic cells mostly depends on expansion conditions rather than isolation methods. They can be expanded using standard culture media composed of a basal medium, supplemented with serum, antibiotics and antimycotics. Some groups use additional supplements including epidermal growth factor (EGF) (Miki et al., 2007; Stadler et al., 2008; Niknejad et al., 2012; Miki et al., 2016) or specific commercial media like endothelial growth medium-2 (Stadler et al., 2008). Although fetal bovine serum (FBS) remains the gold standard in hAMC cultures, efforts have been made to substitute FBS by human alternatives, human platelet lysate or human serum (Wolbank et al., 2010), or even to find serum-free media compositions (Evron et al., 2011; Pratama et al., 2011; Niknejad et al., 2013). hAEC are described to proliferate robustly and display typical cuboidal epithelial morphology between passage 2 and 6 before proliferation ceases (Terada et al., 2000; Ochsenbein-Kölble et al., 2003; Miki et al., 2005) and do not proliferate well at low densities (Parolini et al., 2008). hAEC in culture have been reported to undergo morphological changes from epithelial to fibroblast-like appearance (Portmann-Lanz et al., 2006; Bilic et al., 2008; Stadler et al., 2008; Pratama et al., 2011). These changes are most likely due to epithelial to mesenchymal transition being driven by autocrine transforming growth factor (TGF) beta1 production (Alcaraz et al., 2013) or by external addition of TGF beta1 (Roy et al., 2015). Expansion of hAMSC is possible for at least 5 passages without morphological alterations (Alviano et al., 2007; Bilic et al., 2008; Stadler et al., 2008; Insausti et al., 2010; Lisi et al., 2012). Some groups have even kept hAMSC in culture for 15 to 20 passages before reaching senescence (Tamagawa et al., 2007; Manochantr et al., 2010). Moreover, first-trimester hAMSC have been shown to proliferate better than third-trimester cells (Portmann-Lanz et al., 2006).

#### Human Amniotic Membrane Epithelial Cells (hAEC)

The hAM epithelium is the cellular layer directed towards the fetus and in touch with the AF (Figure 5). hAEC locate in all subregions of the hAM, and hence can be subdivided at least into human reflected amniotic membrane epithelial cells (hRAEC) and human placental amniotic membrane epithelial cells (hPAEC).

Generally, isolated primary hAEC are heterogeneous for their expression of surface antigens and also show varying differentiation capabilities (Centurione et al., 2018), indicating a heterogeneous population [reviewed by Miki and Strom (2006)]. Adding to the heterogeneity is the fact that amnioblasts, derived from the epiblast, may differentiate randomly in the course of embryonic development, and may retain in some cases stem cell properties [reviewed by Miki and Strom (2006)]. Cultured hAEC have been reported to undergo morphological changes from epithelial to fibroblast-like appearance (Portmann-Lanz et al., 2006; Bilic et al., 2008; Stadler et al., 2008; Pratama et al., 2011). These changes are most likely due to epithelial-mesenchymal transition being driven by autocrine TGF beta-1 production (Alcaraz et al., 2013) or by external addition of TGF beta-1 (Roy et al., 2015). Surface marker expression of hAEC has been described to change during culture. Freshly isolated hAEC are positive for CD29, CD49c, CD73, CD166, and stage-specific embryonic antigen (SSEA)-4, TRA-1-60, TRA-1-81 (Centurione et al., 2018), whereas CD13, CD44, CD49e, CD54, CD90, and CD105 increase during the first few passages (Miki et al., 2005; Bilic et al., 2008; Stadler et al., 2008). Furthermore, hAEC at passages (P) 2-4 are positive for CD9, CD10, CD24, CD49e, CD49f, CD140b, CD324 (E-cadherin), CD338 (ABCG2), HLA-A,B,C, SSEA-3, and STRO-1 (Miki et al., 2005; Miki and Strom, 2006; Bilic et al., 2008; Parolini et al., 2008; Magatti et al., 2015; Roy et al., 2015). CD117 (c-kit) and CCR4 (CC chemokine receptor) are demonstrated to be either negative or expressed on a few cells at very low levels (Miki and Strom, 2006; Banas et al., 2008; Bilic et al., 2008; Miki, 2011).

hAEC express markers that are otherwise found exclusively in undifferentiated embryonic stem cells, embryonic carcinoma, and embryonic germ cells (Figure 5A), (Miki et al., 2010). In contrast to embryonic stem cells, hAEC do not express telomerase, are not tumorigenic, and do not become aneuploid (Miki et al., 2005). Octamer-binding transcription factor (Oct)-4 belongs to the POU family of transcriptional regulators and is a transcription factor for maintaining pluripotency and the ability of self-renewal (Rosner et al., 1990; Schöler et al., 1990; Pesce et al., 1998; Lee et al., 2006). Early in development, undifferentiated epiblast cells are positive for Oct-4 and at gastrulation, with stem cell differentiation, Oct-4 is downregulated in somatic cells and only maintained in primordial germ cells [(Pesce et al., 1998); reviewed by Niwa et al. (2000)]. Miki and colleagues proposed that since most of the freshly isolated hAEC express Oct-4, they retain the pluripotency of the undifferentiated epiblast [reviewed by Miki and Strom (2006)]. Interestingly, Oct3/4 appears expressed in all the regions of hAM although at a higher level in the reflected and peripheral regions (Centurione et al., 2018). Oct-4 found in differentiated tissue remains a matter of debate, as two splice variants, Oct-4A and Oct-4B exist. Oct-4A seems to play a role for pluripotency, whereas Oct-4B could be non-functional (Cauffman et al., 2006; Lee et al., 2006). SSEAs are glycolipids that play a role in the compaction process of embryogenesis (Gooi et al., 1981). Compared to term, cells isolated from first trimester hAM express significantly higher levels of cell surface markers TRA-1-60 and TRA-1-81 (Izumi et al., 2009), while in the intact epithelial layer of hAM at term only scattered cells are positive for TRA 1-60 and TRA-1-81, and weakly positive for SSEA-4, and these are surrounded by cells negative for stem cell markers. This may be an indication for the existence of a stem cell “niche”, with a highly specific microenvironment that helps to maintain the stem cell state (Miki et al., 2010).

hAEC in different regions of the hAM may differ in their functional status, such as metabolic and secretory activity (Banerjee et al., 2015; Lemke et al., 2017; Banerjee et al., 2018); however, in relation to surface markers no differences in CD324, CD326, CD73, and SSEA-4 and TRA-1-60 expression have been found (Centurione et al., 2018). Concerning mesenchymal markers, hAEC of all hAM regions have shown absence or poor expression of CD90, CD105, CD146, CD140b, and CD49a.

#### Human Amniotic Membrane Mesenchymal Stromal Cells (hAMSC)

Human amniotic membrane mesenchymal stromal cells are embedded in the rich extracellular matrix of the hAM (Figure 5) and after isolation they confirm the previously reported MSC minimal criteria (Parolini et al., 2008). Furthermore, staining of the intact hAM confirmed that hAMSC lack markers associated with pluripotency, such as TRA-1-60 and TRA-1-81 (Miki et al., 2010). However, the pluripotency markers SSEA-3 and SSEA-4 were reported to be positive (Kim et al., 2007). So far, no phenotype differences have been described for hAMSC originating from different hAM regions, namely the human reflected amniotic membrane mesenchymal stromal cells (hRAMSC) and human placental amniotic membrane mesenchymal stromal cells (hPAMSC).

### Human Chorion (Chorionic Membrane) (hCM)

The chorionic membrane (hCM) represents the fetal component of the placenta. Cells from the hCM are generally referred to as human chorionic membrane cells (hCMC). It is formed by extraembryonic mesoderm and the two layers of trophoblast (syncytiotrophoblast and cytotrophoblast) that surround the embryo and other membranes. The chorionic villi emerge from the entire chorion during the first phases of embryonic development leading to the formation of the primitive placenta, whereas later on they reach the highest level of development and branching in correspondence of the chorion frondosum which rises from the chorionic plate (hCP) and which forms the definitive placenta together with the basal part of the maternal decidua. The rest of the chorion, where villi have turned atrophic, becomes smooth and subtle, does not take part at the formation of the placenta and is called chorion laeve (hCL).

Very early in pregnancy, the chorion of the human placenta starts as a double layer of trophoblast with the syncytiotrophoblast covering the early intervillous space and a layer of cytotrophoblast (hCTB) in second row, proliferating, differentiating and maintaining the syncytiotrophoblast (hSTB) via syncytial fusion. At the end of the second week after fertilization (day 12 p.c.) a new layer of extraembryonic mesenchymal cells (hCMSC) develops and covers the cytotrophoblast towards the embryonic side. Thus, the mesenchymal cells are added as a new border to the original blastocyst cavity without a specific epithelial coverage. At that time of gestation, the amniotic sac is still very small and not in contact with the chorion.

Since first development of placental villi takes place all over the surface of the chorion, the chorion frondosum, i.e., the part of the chorion where placental villi are found, is not restricted to a specific part of the chorion. At this time of placental development, the entire chorion is chorion frondosum and the placenta proper develops as a ball-shaped organ. Only at the end of the first trimester, with the onset of maternal blood flow into the placenta, villous tissues at the abembryonic part of the chorion regress, leading to a smooth chorion, the chorion laeve. The remaining part of the chorion frondosum develops into the chorionic plate covering the placenta proper towards the fetus until delivery. Hence, with the beginning of the second trimester, the placenta changes its shape towards a disk-shaped organ.

#### Human Chorionic Mesenchymal Stromal Cells (hCMSC)

In literature, there are descriptions of the general features and characteristics of mesenchymal cells from term chorion, not differentiating between chorionic plate (hCP-MSC) and chorion laeve (hCL-MSC). Therefore, this paragraph displays the general features of chorionic mesenchymal stromal cells (hCMSC), not differentiating between the two sites.

Several publications have dealt with various isolation methods of placental mesenchymal stromal cells and the comparison of these cells isolated from different placental sites, with the intention to find the most suitable ones. Isolation techniques vary from explant methods (Konig et al., 2015; Wu et al., 2018; Ma et al., 2019; Yi et al., 2020), enzymatic methods (Soncini et al., 2007; Kim et al., 2011; Yamahara et al., 2014; Kwon et al., 2016; Sardesai et al., 2017; Araújo et al., 2018; Ventura Ferreira et al., 2018; Chen et al., 2019; Yi et al., 2020), or the combination of both (Huang et al., 2019). Isolation methods using a modified explant culture technique combined with enzymatic treatment achieved higher cell yield and better proliferative capacity than conventional explant cultures (Huang et al., 2019). hCMSC confirm the already reported MSC morphology, phenotype, and differentiation potential (Dominici et al., 2006; Parolini et al., 2008).

### Human Trophoblast (hTB)

During early development of the embryo, the very first cell lineage of the human is formed. At around day four after fertilization, the blastocyst develops from the morula by differentiation of a cell layer, the human trophectoderm (hTED). This is the forerunner of all trophoblast cells and layers throughout pregnancy. At the time of implantation, around day 6-7 after fertilization, trophectoderm cells in contact with the embryoblast attach to the uterine epithelium. Cells in direct contact with the epithelium fuse and generate the first trophoblast cell type, the syncytiotrophoblast (hSTB) that penetrates through the uterine epithelium. This first multinuclear primitive syncytium is the first invasive placental component that expands into the maternal compartment. The other trophoblast cells remain as undifferentiated human mononuclear cytotrophoblast (hCTB) cells, characterized by an epithelium-like phenotype with cuboidal cell shape (Huppertz, 2008; James et al., 2012; Knöfler et al., 2019).

Three weeks after fertilization placental villi are fully developed. The hSTB has differentiated into the outermost cover of placental villi, now serving as the epithelial layer in direct contact with maternal blood. Below the hSTB, proliferative hCTB make up the second epithelial layer of the placental villi. The villous cytotrophoblast (hVCTB) maintains the hSTB by continuous proliferation, differentiation and fusion with the hSTB (Huppertz, 2008). With the further differentiation and fusion processes of the hVCTB with the pre-existing syncytium the outer multinuclear syncytiotrophoblast layer is maintained (Aplin, 2010), providing the nutritional and oxygen uptake route to the developing embryo and fetus, and secreting hormones to maintain pregnancy, such as human chorionic gonadotrophin (hCG) and placental lactogen (Aplin, 2010).

The syncytiotrophoblast is a multinucleated and polar layer without lateral cell borders, not showing any proliferative activity. The syncytiotrophoblast only has an apical membrane facing maternal blood and a basal membrane in contact to the underlying villous cytotrophoblasts (Figure 3). The maintenance of this syncytial layer is completely dependent on the fusion of villous cytotrophoblast cells throughout pregnancy (Huppertz, 2008).

Trophoblastic cell columns at the tips of anchoring villi attached to the maternal decidua give rise to extravillous trophoblast (hEVT) cells. Proliferative proximal cell column trophoblast (hpCCT) sitting on its basal lamina represents the progenitor cell pool of differentiated hEVT. In the distal cell column, the cells differentiate into non-proliferative distal cell column trophoblast (hdCCT). Some of these cells undergo polyploidization and senescence upon differentiation into hEVT (Velicky et al., 2018). In general, the entire pool of extravillous trophoblast is derived from the cell column trophoblast.

The cell column trophoblast undergoes a multi-step differentiation process with epidermal growth factor receptor-positive (EGFR+) hpCCT, characterized by high proliferative activity. These cells further differentiate into non-proliferating human leukocyte antigen G-positive (HLA-G+) hdCCT, ready to invade the decidua. HLA-G is expressed in all extravillous trophoblast (King et al., 1996). EGFR is expressed by hVCTB and proliferative hpCCT, but absent from hEVT, which specifically up-regulates ERBB2 (Fock et al., 2015).

hEVT can be found at various sites of the feto-maternal interface, such as the chorion laeve, the chorionic plate, cell islands within the villous tissues, and in the uterus at the site of the placental bed. Most of these cells invade into the maternal uterine stroma and the inner third of the myometrium as interstitial extravillous trophoblast (hiEVT) (Huppertz, 2019). From the interstitial subtype of hEVT a number of further subtypes develop and invade into all luminal structures of the placental bed: endovascular extravillous trophoblast (hvasEVT) invades into all maternal vessels of the placental bed in the uterus. They can be further subdivided into trophoblast cells invading into arteries, endoarterial extravillous trophoblasts (hartEVT) and trophoblasts invading into uterine veins, endovenous extravillous trophoblast (hvenEVT) (Moser et al., 2017). Interstitial trophoblast also further invades into uterine glands as endoglandular extravillous trophoblast (hglaEVT) and into uterine lymph vessels as endolymphatic extravillous trophoblast (hlymEVT), (Moser et al., 2010, 2015, 2017, 2018a; Windsperger et al., 2017). The invasion of hiEVT is limited and is halted after reaching the inner third of the myometrium. In the course of gestation hiEVT differentiates from invasive cells into large polyploid cells or fuses to generate multinucleated trophoblast giant cells.

Trophoblast stem cells (hTSC) are the progenitors of the differentiated hCTB in the placenta. They have been isolated from both the chorionic membrane and villous tissue of the placenta [reviewed by Gamage et al. (2016)]. The sources of hTSC or trophoblast progenitor cells (hTPC) are the blastocyst and early first trimester placental tissue. Only recently *in vitro* studies succeeded in deriving hTSC from blastocysts and primary hCTB preparations (Okae et al., 2018), as well as from first trimester placentas using 3D organoid cultures (Haider et al., 2018; Turco et al., 2018; Sheridan et al., 2020). A reason for past failures is the lack of suitable culture conditions (Nursalim et al., 2020), promoting human trophoblast self-renewal and ongoing *in vitro* proliferation of trophoblast cells after isolation from the human placenta. Thus, characteristic stem cell markers for each trophoblast subpopulation have been identified to overcome this problem, such as CDX2 (Caudal Type Homeobox 2), which is abundantly expressed in early first trimester, but becomes rapidly downregulated and restricted to individual hVCTB towards the end of the first trimester (Horii et al., 2016; Soncin et al., 2018). TEA Domain Transcription Factor 4 (TEAD4), E74 like ETS Transcription Factor 5 (ELF5) and transformation-related protein 63 (TP63) are expressed among the hVCTB population of the first trimester human placenta. Transcription Factor AP-2 Gamma (TFAP2C) and GATA Binding Protein 3 (GATA3) are widely expressed across all trophoblast cell types (Lee et al., 2016). Molbay et al., revealed that hTPC isolated from term placenta were positive for the trophoblast stem cell markers CDX2 and Eomesodermin (EOMES) in 92.5% and 92.7% of cells, respectively (Molbay et al., 2018). Interestingly, the investigation of vascular endothelial growth factor (VEGF), VEGF-Receptor 1 (R1), and VEGF-Receptor 2 (R2) at protein and mRNA levels in comparison with human umbilical vein endothelial cells (HUVEC), revealed that hTPC have higher levels of VEGF and VEGFR1 transcripts (Molbay et al., 2018). Soluble forms of VEGF and VEGFR1 were detected in supernatants of hTPC. In 2016, Genbacev et al., suggested that integrin alpha 4 (ITGA4) was the highest expressed factor in trophoblast stem/progenitor cells isolated from term placenta (Genbacev et al., 2016). Therefore, a high level of ITGA4 expression on the surface of the cells may be used to identify hTPC from term placental tissues.

Interestingly, a recent study used trophoblast organoids to generate hTSC (Sheridan et al., 2020). They demonstrate that they can be established within 2-3 weeks, passaged every 7–10 days, and cultured up to one year. Importantly, the authors found that hTSC resembled the villous placenta in their transcriptomes and production of placental hormones. Another group demonstrated that TSC can be isolated from mice using anti-CD117 micro-beads from embryonic day 18.5 mouse placentas (Hou et al., 2020). Furthermore, as mentioned above, the isolation of hTSC from blastocysts has previously been demonstrated (Okae et al., 2018), while others have shown that naïve human pluripotent stem cells (hPSCs) can be used to establish hTSC (Dong et al., 2020).

### Human Chorionic Plate (hCP)

The chorionic plate (hCP) of the human placenta is built up of layers of two different origins, chorion and amnion. The amnion is located towards the fetus and towards the intervillous space the chorion frondosum is found, from which placental villi grow into the trophoblast lacunae. Also, within the chorionic plate the amniotic membrane is an avascular tissue.

The chorion frondosum differs from the chorion laeve in a variety of aspects. It is a spongy layer with few mesenchymal cells and clefts in direct contact with the amniotic membrane. This layer is followed by a thicker layer of compact mesenchyme, in which the chorionic plate blood vessels from the fetus to the placenta (and vice versa) are located. This layer ends with a rudimentary basement membrane towards the intervillous space. At this site, remnants of the complete layering of the chorion early in pregnancy can be found, including small parts of the syncytiotrophoblast and nests or single extravillous trophoblast cells. Since the cytotrophoblast cells are located outside the villous part of the placenta, they are termed extravillous trophoblast. Also, throughout pregnancy more and more fibrinoid covers the surface of the chorionic plate in direct contact with maternal blood.

#### Human Chorionic Plate Cells (hCPC)

**FIGURE 6:**
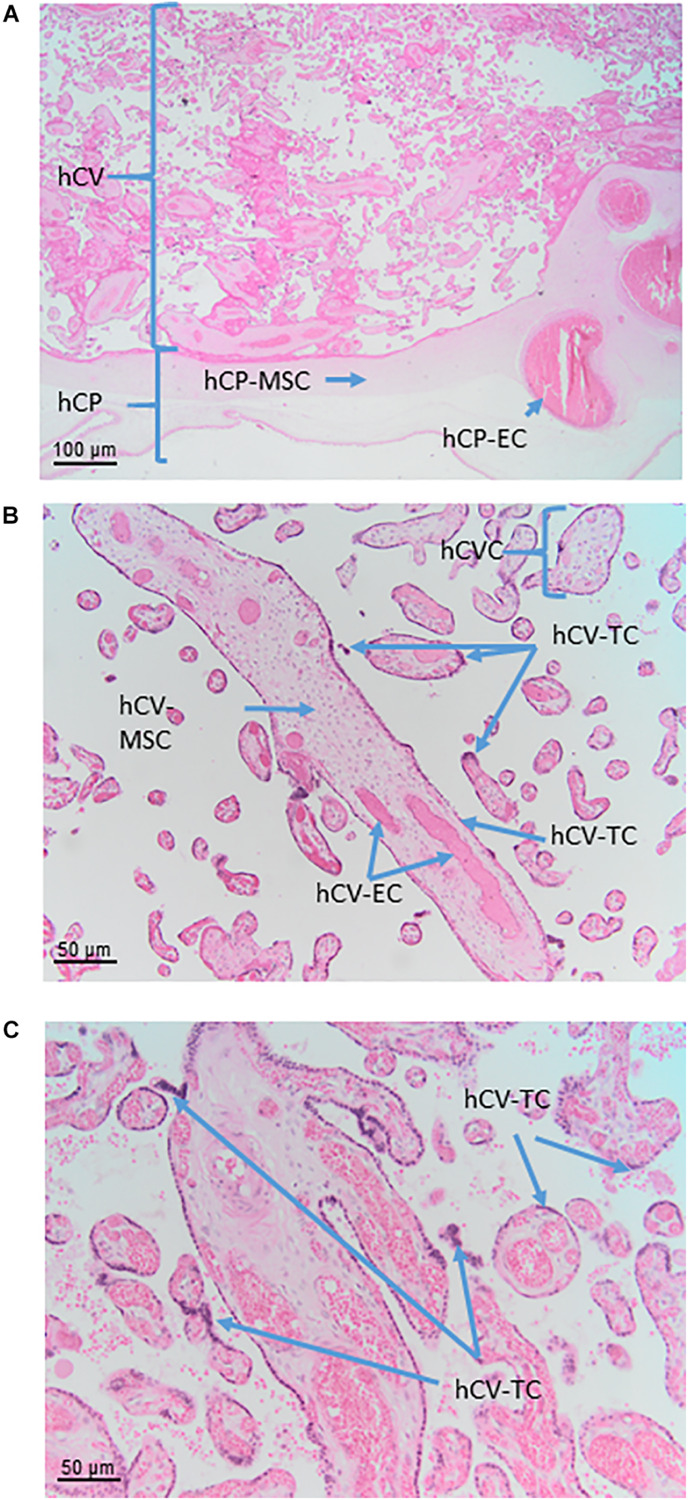
Cell populations from chorionic plate and chorionic villi. Histological images of human chorionic plate (hCP) and chorionic villi (hCV). Haematoxylin-eosin staining. **(A)** At low magnification (10x) the structure of hCP and hCV is appreciable. hCP-MSC: human chorionic plate mesenchymal stromal cells; hCP-EC: human chorionic plate endothelial cells. **(B,C)** At higher magnification (20x) cell populations present in the chorionic villi are more appreciable. hCVC, human chorionic villi cells; hCV-EC, human chorionic villi endothelial cells: hCV-MSC, human chorionic villi mesenchymal stromal cells; hCV-TC, human chorionic villi trophoblast cells.

The cells that are located in the chorionic plate belong to the amnio-chorionic membrane as well as to the chorion frondosum (Figure 6A). The cells of the amniotic membrane are described elsewhere in the text, while the cells of the chorion frondosum are described here. They include stromal mesenchymal cells, vessel-related mesenchymal cells and endothelial cells as well as extravillous trophoblasts.

#### Human Chorionic Plate Mesenchymal Stromal Cells (hCP-MSC)

Some studies describe that hCP-MSC are devoid of maternally derived MSC in contrast to villus-derived MSC and decidua-derived MSC (Wu et al., 2018; Huang et al., 2019). However, several research groups have observed that *in vitro* cell culture passaging of MSC isolated from the chorionic plate and chorionic villi led to a high risk of overgrowth by maternal MSC of the placental decidua (Soncini et al., 2007; Sardesai et al., 2017). This discrepancy could be explained by the use of different isolation methods and cell culture media. Sardesi et al., showed that chorion-derived MSC cultures rapidly became composed entirely of maternal cells when they were cultured in the standard culture medium with serum as fetal MSC did not grow readily under these conditions in contrast to maternal MSC (Sardesai et al., 2017). The authors focused on two key parameters to keep the fetal chorionic MSC culture-free of maternal cells: (1) a careful dissection of fetal tissue from the central cotyledonary core that helps remove the majority of maternal cells during isolation, (2) culture of the fetal chorionic MSC in endothelial growth medium that propagates their proliferative activity and suppresses maternal overgrowth. In line with this, Huang et al., reported that MSC serum free media did not completely prevent maternal contamination when compared to endothelial growth medium supplemented with serum (Huang et al., 2019). Interestingly, hAMSC cultured in endothelial growth medium have a strikingly higher proliferative activity than those cultured in standard medium with serum (Konig et al., 2012).

Human chorionic plate mesenchymal stromal cells express typical MSC markers (Dominici et al., 2006) and a few studies have reported the expression of pluripotency related markers, such as SOX2 and SSEA4, Oct-4 and Nanog, but this is controversially discussed (Cauffman et al., 2006; Lee et al., 2006; Liedtke et al., 2007, 2008). Interestingly, hCP-MSC also express HLA-G, implicated in their immunomodulatory effects and show higher CD106 expression compared to MSC derived from umbilical cord, amnion, and decidua parietalis (Kim et al., 2011; Wu et al., 2018).

#### Human Chorionic Plate Mesenchymal Stromal Cells Derived From Blood Vessels (hCP-MSC-bv)

Human chorionic plate mesenchymal stromal cells derived from blood vessels were isolated as explant cultures from blood vessels of the chorionic plate and cultured in endothelial growth medium (Konig et al., 2015). hCP-MSC-bv seem to support angiogenesis to a higher extent than hAMSC, although both cell types express a very similar MSC surface marker profile (Dominici et al., 2006; Parolini et al., 2008). In addition, both cell types are positive for CD49a, and negative for alkaline phosphatase (AP), and mesenchymal stem-cell like antigen-1 (MSCA-1), smooth muscle actin (smA), desmin and von Willebrand Factor (vWf).

#### Human Chorionic Plate Extravillous Trophoblast (hCP-EVT)

Since the cytotrophoblast cells at the surface of the chorionic plate are not located within villous tissues, they are, by definition, extravillous trophoblast cells. They are the remnants of the complete cytotrophoblast layer of the chorion frondosum early in pregnancy. The extravillous trophoblasts of the chorionic plate (hCP-EVT) display a round to polygonal phenotype with hyperchromatic nuclei showing irregular shapes. Although in general the hCP-EVT display the same phenotypic characteristics as the hEVT in the decidua, they are generally smaller and show less variation in their size and shape. They are HLA-G positive and do not display any signs of proliferation, migration or invasion.

### Human Chorionic Villi (hCV)

In the human placenta, the chorionic villi are arranged as villous trees that are connected via a major trunk to the chorionic plate. From the major trunk, a stem villus, multiple branches develop into intermediate villi, finally ending in free-floating terminal villi (Figure 6B).

All chorionic villi of the human placenta share the same general morphological structure. They have a core of mesenchymal cells derived from extra-embryonic mesoderm. The core structure of all villi contains vessels from large arteries and veins in stem villi down to arterioles and venules in intermediate villi and capillaries and sinusoids in terminal villi. The extracellular matrix is composed of a large number of reticular and collagen fibers.

The vessels are surrounded by MSC that show different levels of differentiation from terminal to stem villi. Also, macrophages (positive for markers such as CD68 and CD163) can be found in the villous stroma of chorionic villi. These macrophages, referred to as Hofbauer cells, are derived from two different sources, directly developing from placental mesenchymal cells in the villous stroma or deriving from circulating fetal monocytes.

The outer cover of the chorionic villi is organized as a two-layered epithelium. The inner layer is composed of mononucleated villous cytotrophoblast (hVCTB) cells that rest on a basement membrane separating the trophoblast cells from the villous stroma. The hVCTB cells proliferate and their daughter cells differentiate to finally fuse with the overlying cover, the hSTB, that is a true syncytium that comes into direct contact with maternal blood circulating around the chorionic villi.

#### Human Chorionic Villi Cells (hCVC)

Within chorionic villi, fetal cells build the epithelial layer as well as the villous core of the villi. The surrounding epithelium is built by a continuous layer of syncytiotrophoblast, in direct contact with the maternal blood, and an underlying layer of mononucleated villous cytotrophoblast. Separated by the trophoblastic basement membrane the villous stroma is filled with mesenchymal cells, macrophages, and vessels formed by endothelial cells, pericytes and smooth muscle cells.

#### Human Chorionic Villi Mesenchymal Stromal Cells (hCV-MSC)

MSC of the chorionic villi express MSC markers (Karlsson et al., 2012; Abomaray et al., 2015; Lankford et al., 2015) in accordance with established minimal criteria (Dominici et al., 2006; Parolini et al., 2008). One study also demonstrated the expression of embryonic stem cell markers such as TRA-1-61, TRA-1-80 and SSEA-4 (Yen et al., 2005), although as mentioned previously the expression of pluripotency markers by MSC is widely debated. hCV-MSC differentiate within the maturation of the growing villous tree (Benirschke et al., 2006). This differentiation starts with MSC (vimentin positive) and continues with MSC and reticulum cells (vimentin and desmin positive) and fibroblasts (vimentin, desmin and alpha smooth muscle actin positive), and finally to myofibroblasts (vimentin, desmin, alpha smooth muscle actin and gamma smooth muscle actin positive), (Benirschke et al., 2006).

#### Human Chorionic Villi Trophoblast Cells (hCV-TC)

The villous trophoblast as a tissue can be divided into two types of cells/layers: mononucleated cytotrophoblasts and the multinucleated syncytiotrophoblast. It needs to be stressed here that in a given placenta, there is only one syncytiotrophoblast covering all chorionic villi of that placenta (Figure 6C).

##### Villous cytotrophoblast

The layer of the mononucleated villous cytotrophoblast is the basal and germinative layer of the villous trophoblast compartment. The cells rest on the basement membrane underneath the villous trophoblast layers. Villous cytotrophoblast cells change their morphology during pregnancy. During the first trimester of pregnancy, the cells display a cuboidal shape and form a nearly complete layer. At term, the cells display a flattened phenotype, separated from each other but connected to each other by long cytoplasmic extensions. A subset of the cells proliferates throughout pregnancy, which may point to a small subset of progenitor cells within this layer. Also, some of the cells may display their progenitor status as it has been shown that they can be induced to differentiate towards the extravillous lineage (Baczyk et al., 2009).

##### Villous syncytiotrophoblast

The syncytiotrophoblast is a continuous, multinucleated layer without lateral cell borders. Hence, a single syncytiotrophoblast covers all villi of a single placenta. The apical membrane of the highly polarized syncytiotrophoblast shows microvilli to amplify seven-fold the surface (Burton and Fowden, 2015) for a better uptake of nutrients from maternal blood. As the syncytiotrophoblast is highly differentiated, growth and maintenance of this layer is dependent on continuous fusion with the underlying cytotrophoblast. The absence of expression of class I or II major histocompatibility complex proteins in the apical membrane of the syncytiotrophoblast is important for its immunological protection (Moffett and Loke, 2006).

### Human Chorion Laeve (Chorionic Laeve Membrane) (hCL)

**FIGURE 7:**
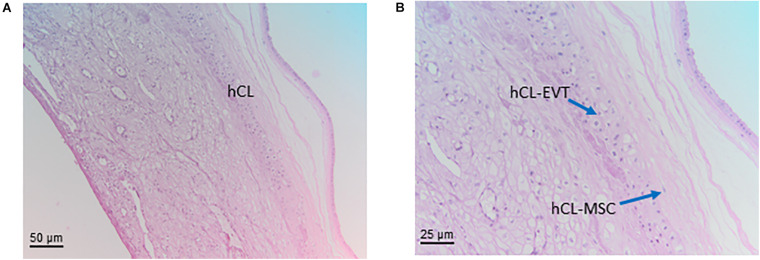
Structure of the chorion laeve. Histological images of human amnio-chorionic membrane (hACM) in correspondence of the chorion laeve (hCL) and the capsular decidua (hCD). Haematoxylin-eosin staining. **(A)** At low magnification (20x) a general overview of this portion of the amnio-chorionic membrane, which has a smooth appearance due to the absence of chorionic villi, is appreciable. **(B)** At higher magnification (40x) it is possible to appreciate more cell populations present in the chorion laeve including extravillous trophoblast (hCL-EVT) and mesenchymal stromal cells (hCL-MSC).

The chorion laeve of the human placenta (hCL, Figure 7A) develops at the end of the first trimester when the ball-shaped placenta develops into a disk-shaped organ. The parts of the early chorion frondosum that are not integrated into the newly developing disk-shaped placenta show degeneration of their placental villi, resulting in the smoothing of this part of the chorion. This is why the chorion laeve is also termed smooth chorion, or fetal membrane. As the amniotic membrane develops into a layer covering the whole placenta, the chorion laeve also contains the amniotic membrane as a cover towards the fetus.

The chorionic part of the chorion laeve is organized into a thin compact stromal layer, densely packed with collagen fibers and containing only few scattered mesenchymal cells, and a fibroblast layer with mesenchymal stromal cells (hCL-MSC, Figure 7B) including fibroblasts and fewer myofibroblasts and macrophages. The chorionic layer of the chorion laeve ends with a basement membrane that separates the mesenchyme from the extravillous trophoblast of the chorion laeve (hCL-EVT). The hCL-EVT layer does not show signs of proliferation, but only displays fully differentiated extravillous trophoblastst in a term placenta. At some sites and at the end of pregnancy, atrophic villi can be detected from the time of villous degeneration. Their stroma can still be intact while vessels are missing. Such “ghost” villi are surrounded by hCL-EVT.

Towards the uterus in touch with the chorion laeve is the only maternal layer of this part of the placenta, the capsular decidua (hCD). This layer includes decidual cells as well as some smaller vessels plus mobile cells such as macrophages and lymphocytes. It needs to be stressed that at the time of delivery the capsular decidua or decidua capsularis is firmly attached to the parietal decidua or decidua parietalis and, hence, some parts of the decidua parietalis may be associated with the decidual layer of the chorion laeve after delivery.

#### Human Chorion Laeve Mesenchymal Stromal Cells (hCL-MSC)

Human chorion laeve mesenchymal stromal cells are plastic adherent cells that follow the minimal phenotype and differentiation criteria of the consensus paper by Parolini et al., (Parolini et al., 2008). In addition, one study also suggested that hCL-MSC can differentiate *in vitro* into cardiomyocytes and express genes associated with heart morphogenesis and blood circulation including serotonin receptor B2 (HTR2B) (Kwon et al., 2016). hCL-MSC also display strong immunomodulatory properties (Chen et al., 2019).

Human chorion laeve mesenchymal stromal cells have also been shown to secrete cytokines quite common to MSC, such as Insulin Growth Factor-1 (IGF-1), VEGF, Hepatocyte growth factor (HGF), basic fibroblast growth factor (bFGF), Angiopoietin 1 (Ang-1), and TGF, with partly contradictory data about lower or higher secreted levels compared to hMSC derived from amnion, umbilical cord, chorionic villi or decidua parietalis (Yamahara et al., 2014; Yi et al., 2020).

#### Human Chorion Laeve Extravillous Trophoblast Cells (hCL-EVT)

The extravillous trophoblast of the chorion laeve (hCL-EVT) displays a round to polygonal phenotype and irregularly formed nuclei that are hyperchromatic. Also, the hCL-EVT cells are generally smaller than the hEVT in the placental bed. Interestingly, although hCL-EVT are HLA-G positive, they do not show signs of invasiveness, different to their counterparts in the placental bed. It has been speculated that local factors keep these cells in a non-invasive state. Isolation of hCL-EVT has been performed and published (Gaus et al., 1997).

### Human Umbilical Cord (hUC)

In placental mammals, the umbilical cord (UC) (also called *funiculus umbilicalis*) is a conduit that connects the placenta to the developing embryo/fetus and is responsible for exchange of nutrients and gasses during gestation. The human umbilical cord is formed when the body stalk (including allantois) and the vitelline duct (also called ductus omphalo-entericus) deriving from the yolk sac plus the umbilical coelom are enveloped by the spreading amnion between the fourth and the eighth week. During the third month of gestation, numerous elements degenerate: the vitelline duct (it can remain in the form of a Meckel’s diverticulum); the allantois (it is obliterated to form the median umbilical ligament); the vitelline circulation system in the extra-embryonic region; the umbilical coelom, which clumps and disappears. In humans, the umbilical cord is approximately 50 cm long and 2 cm in diameter at term and is normally attached in the middle of the placenta.

**FIGURE 8:**
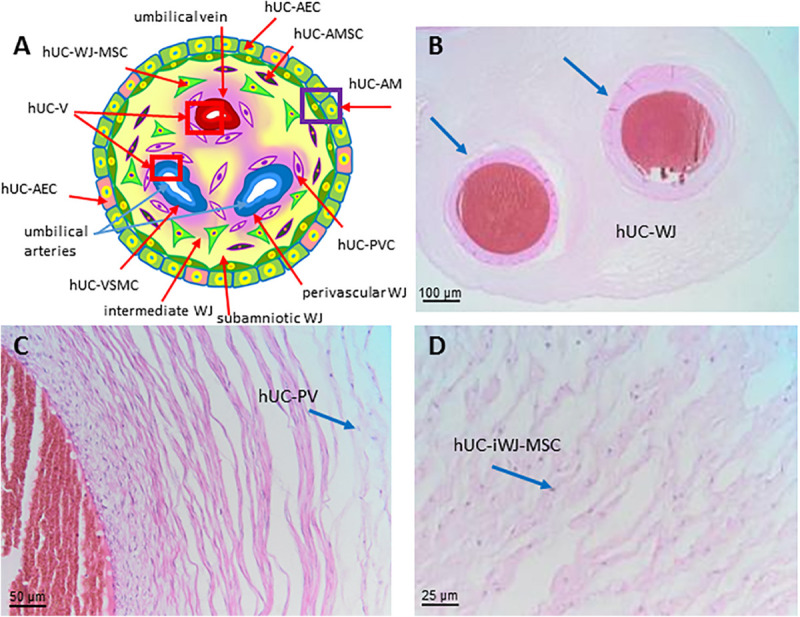
Structure and cell populations from the umbilical cord. **(A)** Schematic structure of the human umbilical cord showing the presence on the surface of the amniotic membrane (hUC-AM) made of hUC-AEC and hUC-AMSC and the different regions of Wharton’s jelly (subamniotic, intermediate and perivascular). hUC-WJ-MSC, human umbilical cord Wharton’s jelly mesenchymal stromal cells; hUC-PVC, human umbilical cord perivascular cells; hUC-V, human umbilical cord vascular region; hUC-VSMC, human umbilical cord vascular smooth muscle cells. Histological images (Haematoxylin-eosin) of **(B)** hUC at low magnification (10x) showing the presence of two umbilical arteries (light blue arrows) surrounded by human umbilical cord Wharton’s jelly (hUC-WJ), **(C)** umbilical artery at higher magnification (20x) showing the presence of human umbilical cord perivascular cells (hUC-PV), **(D)** intermediate region of Wharton’s jelly showing at higher magnification (40x) the presence of numerous mesenchymal stromal cells (hUC-iWJ-MSC).

As shown in Figure 8A the structure shows an outer layer of amniotic membrane (human umbilical cord amniotic membrane, hUC-AM) that surrounds a mucoid connective tissue called “Wharton’s jelly” (human umbilical cord Wharton’s jelly, hUC-WJ), (Figure 8B), as Thomas Wharton described it for the first time in 1656. This gelatinous and elastic connective tissue is full of mesenchymal stem/stromal cells (human umbilical cord vascular region, hUC-V) and generally contains three vessels in humans, one vein and two arteries, which carry oxygenated and deoxygenated blood between the placenta and the fetus, respectively. Unlike other vessels of similar diameter, the umbilical vessels display only a tunica intima and media but are devoid of a tunica adventitia (Davies et al., 2017). Currently, the roles of the tunica adventitia (vascular support and some contractile function) are considered to be fulfilled by the “Wharton’s jelly,” which also protects the umbilical vessels from possible mechanical pressure and creasing. The jelly does not contain other blood or lymph vessels and is devoid of nerve supply. The absence of adventitia and other vessels, besides the two arteries and one vein, is not typical of animal models commonly used in research. This should be taken into account when the same methods to extract cells from umbilical cords are employed across species since variation in structure represents a source of variability in the harvested cell population.

According to Mennan et al., 2013 the human umbilical cord contains distinct anatomical regions comprising the vascular region, cord lining, and Wharton’s jelly (Mennan et al., 2013). Nanaev et al. (1997) identified three regions within the human umbilical cord at term, based on the distribution of extracellular matrix proteins and cytoskeletal features of the stromal cells: the sub-amniotic zone, Wharton’s jelly and the combined media and adventitia of the blood vessels (Nanaev et al., 1997). Vieira Paladino et al. (2019) identified three different regions inside Wharton’s jelly: sub-amniotic, intervascular and perivascular regions (Vieira Paladino et al., 2019). Here we propose the following umbilical cord regions and nomenclature taking into account histological characteristics and embryological development: human umbilical cord amniotic membrane (hUC-AM, Figure 8A), corresponding to the cord lining of other classifications, human umbilical cord Wharton’s jelly (hUC-WJ, Figure 8B), including the sub-amniotic and intervascular regions of other classifications, and human umbilical cord vascular region (hUC*-*V), referred to cell populations present within the wall or in the perivascular region of umbilical vein and arteries contained in the umbilical cord (Figures 8A,C).

#### Human Umbilical Cord Cells (hUCC)

The major cellular component derived from the umbilical cord is composed of stromal cells that have a differing range of differentiation potential from mesenchymal to myofibroblast phenotype and can express various levels of cytoskeletal markers such as vimentin, desmin, cytokeratin, and alpha smooth muscle actin (Can and Balci, 2011; Conconi et al., 2011). Considering this heterogeneity, hUCC located near the outer amniotic layer are the most undifferentiated cells, sustaining the presence of mesenchymal stromal cells in the amniotic fluid. Conversely, hUCC close to the vascular zone are more differentiated stromal cells and committed to myofibroblasts (Can and Balci, 2011; Conconi et al., 2011). More recently, the connective tissue, in which these stromal cells reside, has been hypothesized to consist of three different anatomical regions - perivascular, intermediate, and cord lining or sub-amniotic - with distinctive characteristics and specific cellular populations (Davies et al., 2017).

Furthermore, stromal cells within the umbilical cord display different characteristics depending on the region from which they are derived. These peculiar features can be associated with the fact that umbilical cord presents two different embryonic origins: from the connection of stalk and allantoid mesenchyme and from the covering of the amniotic membrane (Subramanian et al., 2015). Furthermore, cell isolation methods are still not standardized and due to the existence of two different approaches (enzymatic and explant-based), there are various degrees of variability for harvesting the cells from the umbilical cord. In particular, enzymatic-based protocols are commonly used for the isolation of hUCC by using collagenase, hyaluronidase, or other proteases. Differently, explant-based isolation procedures are used because of higher cell yield and preserving cell membrane protein integrity (Abbaszadeh et al., 2020).

#### Human Umbilical Cord Mesenchymal Stromal Cells (hUC-MSC)

These cells are discussed under the paragraph of human umbilical cord Wharton’s jelly cells because the connective tissue of the umbilical cord is recognized as Wharton’s jelly (Davies et al., 2017).

#### Human Umbilical Cord Amniotic Membrane Cells (hUC-AMC)

The amniotic membrane lining of the umbilical cord represents a possible source of two perinatal cell types: epithelial cells from the epithelium of amniotic membrane and MSC from the stromal side blended with the Wharton’s jelly (Lim and Phan, 2014).

#### Human Umbilical Cord Amniotic Epithelial Cells (hUC-AEC)

The nature of the covering amniotic epithelium has not been thoroughly investigated. Recent studies have demonstrated that human umbilical cord amniotic epithelial cells (hUC-AEC) share common features with fetal epidermal keratinocytes in terms of the expression patterns of cytokeratins, cell surface markers, and their differentiation potential (Ruetze et al., 2008). Umbilical cord amniotic membrane cultured in modified keratinocyte culture medium yields polyhedral epithelial cells and during primary culture, hUC-AEC cells show a cobblestone appearance characteristic of typical epithelial cells. The proliferation rate at the beginning is relatively slow but then, after the initial clonal growth, it becomes faster and gives rise to large and tightly packed colonies (Lim and Phan, 2014).

Phenotype characterization of hUC-AEC has shown expression of CK8, CD14, and CD19, markers associated to simple epithelium and skin stem cells. Immunophenotype analysis has shown that hUC-AEC are negative for CD45, CD90, CD105, whereas they are positive for CD29, CD44, CD49f, CD166. One study reported that a small fraction of hUC-AEC display stem cell-specific molecules such as SSEA-4 and TRA-1-60 (Huang et al., 2011).

#### Human Umbilical Cord Amniotic Mesenchymal Stromal Cells (hUC-AMSC)

Considering the embryological development of the umbilical cord, the stromal component of the amniotic membrane is completely fused with the connective part of the umbilical cord. For this reason, it is difficult to distinguish and isolate a cell population of amniotic origin with mesenchymal characteristics (consider that during embryonic development the connective tissue of the body stalk and the amniotic membrane form a common umbilical cord connective tissue known as “Wharton’s jelly,” for more details see Carlson (2013). However, several authors have proposed that when amniotic membrane is cultured in modified fibroblast media, spindle shaped fibroblast-like mesenchymal cells can be obtained with plastic adherent properties. Growing to confluence, these cell strains can form colonies, a gross morphological indicator of stem cell identity (Lim and Phan, 2014).

#### Human Umbilical Cord Wharton’s Jelly Cells (hUC-WJC)

There is no consensus on the experimental protocols for isolation of cells from Wharton’s jelly, nor on the anatomical structure of the cord, and particularly that of the zones of Wharton’s Jelly, from which the cells are extracted. As a matter of fact, it is rarely possible to glean from published methods which cells are specifically being cultured in experiments, or more importantly, employed in clinical trials. Thus, there is an urgent need to arrive at a consensus on the anatomical structure of the cord, and particularly that of the zones of Wharton’s Jelly, from which the cells are extracted.

Nonetheless, there is a current consensus that cells isolated from Wharton’s jelly display MSC characteristics (Dominici et al., 2006; Parolini et al., 2008). They can have two distinct morphologies: flat, wide cytoplasmic cells, and slender fibroblast-like cells (Karahuseyinoglu et al., 2007). These two cell populations differ in their cytoskeletal filament content: vimentin (mesenchymal marker) and pan-cytokeratin (ecto-endodermal marker). As shown in Figure 8, the cell population positive for both vimentin and pan-cytokeratin appears flattened and is localized in the perivascular region (type I cell). The cell population with a more fusiform and elongated (type II cell) cytoplasm/morphology is positive only for vimentin, and it is located in the intervascular region.

#### Human Umbilical Cord Wharton’s Jelly Mesenchymal Stromal Cells (hUC-WJ-MSC)

Protocols for the isolation of hUC-WJ-MSC have not been standardized. These cultures can be obtained either by enzymatic digestion or by explant cultures, and both approaches have been argued to be effective only when applied on fresh WJ tissue (Davies et al., 2017).

Despite these limitations, Wharton’s jelly cells derived from the human umbilical cord (hUC-WJC) also follow the minimal phenotype and differentiation criteria of the consensus paper by Parolini et al., (Parolini et al., 2008), and express other mesenchymal common markers such as CD10, CD13, CD29, CD44, CD54, CD73, CD90, CD105, Stro-1, α-smooth muscle actin (αSMA), vimentin, MHC class I molecules (classic HLA-A, -B, and –C, and non-classical HLA-G, -E, and -F), and lack typical hematopoietic and endothelial markers CD14, CD19, CD31, CD34, CD38, CD45 CD66b, CD80, CD86, CD106, CD133, and HLA-DR (Lee et al., 2004; Moodley et al., 2009; Corrao et al., 2013; Wang et al., 2018). The hUC-WJ-MSC immunophenotype has usually been compared to mesenchymal cells derived from adult sources but, in addition, there is some evidence of pluripotent stem cell markers such as TRA-1-60, TRA-1-81, SSEA-1, and SSEA-4 even if the expression levels are significantly lower than in pluripotent cells and strictly depend on isolation procedure and culture conditions (Fong et al., 2011; Musiał-Wysocka et al., 2019).

#### Human Umbilical Cord Sub-Amnion Wharton’s Jelly Mesenchymal Stromal Cells (hUC-saWJ-MSC)

As mentioned above, the cord lining membrane mesenchymal cells are fibroblast-like cells with spindle-shaped morphology (Figure 8A) that meet the minimal criteria of perinatal MSC (Parolini et al., 2008) for their typical adherence capacity and the immunophenotype pattern (Lilyanna et al., 2013). hUC-saWJ-MSC are positive for the mesenchymal cell markers as reported in Parolini et al. (2008), moderately express the embryonic stem cell marker SSEA-4, and they lack hematopoietic and endothelial cell markers (Deuse et al., 2011).

Interestingly, hUC-saWJ-MSC can also display epithelial cell properties due to the expression of cytokeratins CK1, CK7 and CK14, the epithelial cell marker MUCIN1 (CD227), and the epithelial cell-to-cell adhesion molecule CD151 (Kita et al., 2010; Reza et al., 2011).

#### Human Umbilical Cord Intermediate Wharton’s Jelly Mesenchymal Stromal Cells (hUC-iWJ-MSC)

For details on this cell population please refer to the cell population described in the paragraph on hUC-WJ-MSC (Figures 8A,D).

#### Human Umbilical Cord Perivascular Cells (hUC-PVC)

Almost 45% of the cells resident in the Wharton’s jelly reside in the perivascular region (Figure 8C). Umbilical cord perivascular cells have been shown to be positive for platelet derived growth factor-receptor ß (PDGF- Rß) and CD146 (Avolio et al., 2017)and NG2 (Montemurro et al., 2011). hUC-PVC are also positive for MSC markers (Sarugaser et al., 2009; Lv et al., 2014) described in the minimal consensus criteria (Dominici et al., 2006; Parolini et al., 2008).

#### Human Umbilical Cord Vascular Smooth Muscle Cells (hUC-VSMC)

Umbilical cord vessels can be considered a common source of vascular smooth muscle cells (Figure 8A). Human umbilical cord vascular smooth muscle cells (hUC-VSMC) can be obtained either by enzymatic digestion or by explant cultures (Mazza et al., 2016; Thormodsson et al., 2018). *In vitro* culture of human umbilical cord vascular smooth muscle cells (hUC-VSMC) promotes a switch from a contractile (quiescent) phenotype to a more secretive (proliferating) one (Roffino et al., 2012). In many previous studies, the characterization of primary hUC-VSMC has been limited to the expression of the characteristic contractile protein αSMA. Besides αSMA, hUC-VSMC have typical MSC phenotype (Dominici et al., 2006; Parolini et al., 2008) and are positive for smooth muscle myosin heavy chain (SM-MHC), desmin, and vimentin. In contrast to human umbilical cord perivascular cells (hUC-PVC), hUC-VSMC have been reported to be negative for CD10 and display a high expression of SM-MHC (Mazza et al., 2016).

#### Human Umbilical Cord Myofibroblasts (hUC-MF)

The differentiated myofibroblasts represent the functional phenotype of Wharton’s jelly. Mesenchymal cells that comprise the functional myofibroblasts of the stroma and their precursors are found in this unusual connective tissue. These have a fibroblast-like morphology and can be identified in five stages of differentiation based on their sequential and additive expression of vimentin, desmin, αSMA, γ-SMA, and smooth muscle myosin (Nanaev et al., 1997; Davies et al., 2017).

### Blood Vessels of the Human Term Placenta and Umbilical Cord

As mentioned earlier, the umbilical cord comprises two fetal arteries and one fetal vein. The umbilical arteries transport low-oxygenated blood, loaded with metabolic waste products, from the fetus via fetal arteries of the chorionic plate into the placental villous tree, which mainly consists of (1) stem villi with centrally located smaller arteries and veins, and a paravascular capillary network; (2) intermediate villi with arterioles, venules, capillaries; and (3) terminal villi with sinusoids and capillaries. The intervillous space is filled with maternal blood, supplied by afferent maternal spiral arteries penetrating the basal plate and efferent maternal utero-placental veins. The feto-maternal exchange takes place across the vasculo-syncytial membrane of the terminal villi. There, the fetal blood is loaded with oxygen and nutrients and transported via the venous system of intermediate and stem villi, veins of the chorionic plate and the umbilical vein back to the fetus (Figure 1B).

Early *in situ* studies evidenced heterogeneity of the endothelium of the umbilical cord and placental blood vessels (Lang et al., 1993, 1994; Dye et al., 2001). Placental fetal endothelium expresses CD32 and the transferrin receptor, contrary to the endothelium of the umbilical cord and maternal vessels, Bandeiraea simplicifolia (BS-I) lectin heterogeneously stains placental and maternal vessels, but not umbilical cord endothelium. Indoleamine 2, 3-dioxygenase 1 (IDO-1) is absent in endothelial cells of the umbilical cord, but present in endothelial cells of the human term placenta (Blaschitz et al., 2011).

**FIGURE 9:**
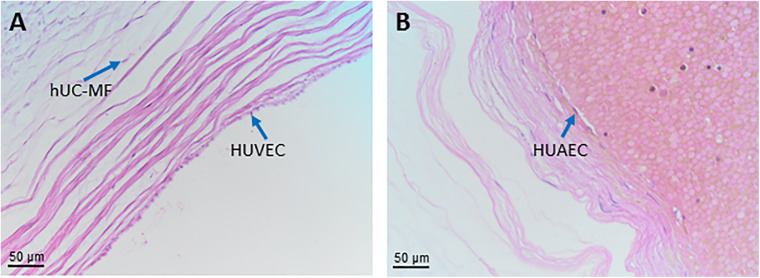
Cells from blood vessels of human umbilical cord. Histological sections of human umbilical vein **(A)** and artery **(B)** at low magnification (20x). Haematoxylin-eosin staining. hUC-MF, human umbilical cord myofibroblasts; HUAEC, human umbilical artery endothelial cells surrounding a lumen containing blood cells; HUVEC, human umbilical vein endothelial cells surrounding an empty lumen.

Endothelial cells have been mainly isolated by enzymatic digestion from the human umbilical vein (HUVEC, Figure 9A), to a smaller extent from umbilical arteries (HUAEC, Figure 9B) and fetal placental vessels (hP-EC). They share typical endothelial characteristics: they express vWF, which is stored in endothelial-specific Weibel-Palade bodies, CD31, CD144, VEGF-R2 and can be stained with Ulex europaeus I lectin that specifically binds to L-fucose residues (Holthöfer et al., 1982; Hormia et al., 1983; Hannah et al., 2002; van Mourik et al., 2002). They take up acetylated low-density lipoprotein (acLDL) (Voyta et al., 1984), form networks on Matrigel and lack expression of hematopoietic markers like CD14, CD15, CD45, the mesenchymal stromal cell marker CD90, and desmin, myosin, and smooth muscle actin. Some endothelial markers change their expression pattern during culture. Especially CD34, a most reliable endothelial marker *in situ*, is only detectable in 20-30% of cultured endothelial cells even in early passages (Müller et al., 2002).

HUVEC, HUAEC and hP-EC show differences in their phenotype, genotype, functionality and DNA methylation profile (Casanello et al., 2014) as summarized in the following sections.

#### Human Umbilical Vein Endothelial Cells (HUVEC) and Human Umbilical Arterial Endothelial Cells (HUAEC)

Human umbilical vein endothelial cells HUVEC and HUAEC have a cobblestone morphology *in vitro*. Specific transcription factors, shear stress, and oxygen levels control the differential expression of arterial- and venous-related genes (e.g., Hey2, EphrinB2, NICD4 and COUP-transcription factor 2 (TF2), respectively). HUAEC express higher levels of plasminogen activator inhibitor-1 (PAI-1), Cx40, *17β*-Hydroxysteroid dehydrogenases (17β-HSD2), and vascular cell adhesion molecule *1* (VCAM-1); and lower levels of vWF and estrogen receptors β when compared to HUVEC. HUVEC and HUAEC differ in their expression of angiotensin converting enzyme, endothelin-1 and endothelial nitric oxide synthase (eNOS) activity (Casanello et al., 2014).

Similar to hP-EC, HUVEC express CD31, CD34, CD105, CD144, PAL-E, Tie-1, Tie-2, VEGF-R1, VEGFR-2 and HLA class I molecules, but are negative for CD36 and CD133, contrary to hP-EC (Sölder et al., 2012). HUVEC express lower levels of angiotensin II, endothelin, and thromboxane, differ in homeobox gene expression, and have a lower cholesterol transport capacity and a lower proliferative response to cytokines when compared to hP-EC (Casanello et al., 2014).

#### Human Placental Endothelial Cells (hP-EC)

Fetal hP-EC have no explicit nomenclature. This is partly caused by different isolation methods, resulting in endothelial cell cultures derived from various vascular regions, or the focus of the respective manuscripts. According to Sölder and colleagues, hP-EC grow either in cobblestone or in swirling pattern (Sölder et al., 2012). They express vWF, UEA-1, HLA-class I, CD31, CD34,CD36, CD51/61, CD54, CD62E, CD105, CD106, CD133, CD141, CD143, CD144, CD146, VEGF-R1, VEGFR-2, EN-4, PAL-E, BMA120, Tie-1, Tie-2, α-tubulin, but are negative for VEGFR-3, LYVE-1, Prox-1, podoplanin, CD14, CD45, CD68, HLA Class II. At the ultrastructural level, hP-EC harbor numerous microvilli, micropinocytic vesicles at their basis, and are rich in intermediate filaments.

#### Human Chorionic Villous Endothelial Cells (hCV-EC) and Human Placenta Microvascular Endothelial Cells (hP-mV-EC)

The terms hCV-EC and hP-mV-EC are often used synonymously, although chorionic villi also comprise macrovascular endothelial cells. Endothelial cells are obtained by enzymatic perfusion of placental vessels (Schütz and Friedl, 1996; Jinga et al., 2000; Lang et al., 2003; Murthi et al., 2007; Lang et al., 2008; Murthi et al., 2008) or mechanical dissection followed by enzymatic digestion and enclosed purification using immunomagnetic beads (Leach et al., 1994; Wang et al., 2004a; Su et al., 2007; Escudero et al., 2008; Sölder et al., 2012; Salomon et al., 2013; Troja et al., 2014; Palatnik et al., 2016; Morley et al., 2018; Gao et al., 2020), or they can be obtained from cultured microvessels after serial sieving of placental villi and subsequent digestion of perivascular cells (Challier et al., 1995; Kacémi et al., 1997). Enzymatic perfusion of a placental lobule leads to endothelial cultures enriched in microvascular endothelial cells (Lang et al., 2003; Murthi et al., 2007; Murthi et al., 2008). Enzymatic perfusion of the placental vasculature via the umbilical vein results in cell preparations, which tend to originate from the venous system of placental stem villi rather than from the microvasculature (Schütz and Friedl, 1996; Jinga et al., 2000; Lang et al., 2003). On the basis of the absent staining with BS-I lectin, several authors suggested the microvascular origin of the isolated cells (Schütz and Friedl, 1996; Jinga et al., 2000). However, deeper in the chorionic villi, especially in intermediate and terminal villi, which contain the major part of the microvasculature (Kaufmann et al., 1985), the endothelium of blood vessels becomes increasingly reactive with BS-I (Lang et al., 1994). Thus, the cells isolated (Schütz and Friedl, 1996; Jinga et al., 2000) may predominantly be derived from medium-sized veins of stem villi and by definition are not of ’microvascular origin’, a terminology, which is restricted to arterioles, venules and capillaries. These venous placental endothelial cells share some similarities with microvascular placental endothelial cells like spindle-shaped morphology, growth in swirling patterns, and network formation at post-confluent state (Challier et al., 1995; Kacémi et al., 1997). A recent study by Gao et al., revealed that isolating clonal ECFCs from human early gestation chorionic villi (CV-ECFCs) of the placenta by enzymatic digestion and isolation by CD31-magnetic beads have the potential for fetal tissue engineering (Gao et al., 2020).

#### Human Chorionic Plate Endothelial Cells (hCP-EC)

Enzymatic perfusion of chorionic arterial or venous blood vessel segments leads to endothelial cultures of defined origin (Lang et al., 2008). *In vitro*, human placental arterial endothelial cells (hPA-EC) are polygonal cells with a smooth surface and grow in loose arrangements and forming monolayers with cobblestone morphology. They express artery-related genes (hey-2, connexin 40, depp) and more endothelial-associated genes than human placental venous endothelial cells (hPV-EC). VEGFs induce a higher proliferative response on hPA-EC, whereas placental growth factors (PlGFs) are only effective on hPV-EC. *In vitro*, hPV-EC are spindle-shaped cells with numerous microvilli at their surface. They grow closely apposed to each other, form fibroblastoid swirling patterns at confluence and have shorter generation and population doubling times than hPA-EC. hPV-EC over-express development-associated genes (gremlin, mesenchyme homeobox 2, stem cell protein DSC54), and show an enhanced adipogenic and osteogenic differentiation potential unlike hPA-EC (Lang et al., 2008). These data provide evidence for a juvenile venous and a more mature arterial phenotype of hCP-EC. The high plasticity of hPV-EC may reflect their role as tissue-resident endothelial progenitors during embryonic development with a possible benefit for regenerative cell therapy (Lang et al., 2008). A comparison of the genome-wide DNA methylation profile in hPA-EC and hPV-EC show that venous endothelial cells present lower levels of global methylation compared to hPA-EC (Joo et al., 2013). hCP-EC were shown to express IDO-1 unlike HUVEC (Blaschitz et al., 2011). The DNA methylation status of NOS3 (eNOS) and ARG2 (arginase-2) promoters by pyrosequencing suggest the presence of site-specific differences between hPA-EC, HUAEC and HUVEC (Casanello et al., 2014)

### Human Amniotic Fluid (hAF)

From the second to the fourth week of gestation, the amniotic fluid (hAF) gradually increases and separates the cells of the epiblast (embryo) from the amnioblasts surrounding the embryo in the newly formed amniotic cavity. The hAF allows for fetal movements and growth inside the uterus. It also allows for the exchange of different nutrients and chemicals between the fetus and the mother (Fauza, 2004; Dobreva et al., 2010).

Human AF cells (hAFC) can be isolated during the three trimesters of gestation (Di Trapani et al., 2015; Schiavo et al., 2015; Spitzhorn et al., 2017), and have a heterogeneous origin since they come from the urinary and pulmonary secretions of the fetus, the skin, the digestive tract and the amniotic membrane (Underwood et al., 2005) The phenotypic studies characterize the cells into many shapes, from round to squamous, and different sizes, ranging from 6 to 50 μm in diameter (De Coppi et al., 2009), all of which grow in adhesion. It is conceivable that different methods of hAF cell isolation give rise to cells with different morphology. The “plastic adherence” is the most simple method used to obtain cells from the hAF (Steigman and Fauza, 2007), whereby fibroblast-like cells prevail. The “immunoselection” method is based on the idea that the stem cell population within hAF, that is present at a very low percentage, can be selected using CD117 (or c-Kit) which is the most commonly used antigen that identifies the hAF stem cell population referred to as human amniotic fluid stem cells (hAFSC) (De Coppi et al., 2007; Pozzobon et al., 2013). As described below, the capability of this subpopulation to differentiate according to the different *in vitro* and *in vivo* stimuli suggests their stem cell origin (Ditadi et al., 2009; Piccoli et al., 2012). In 2004 a method based on “two step culture” was also proposed where non-adherent cells from the hAF are first seeded in media without serum, followed by a second seeding in petri dishes (Tsai et al., 2004).

#### Human Amniotic Fluid Cells (hAFC)

Human amniotic fluid cells are a heterogeneous population that can be classified into three groups according to their molecular, morphological, and growth characteristics (Prusa and Hengstschlager, 2002; Pipino et al., 2015): (1) epithelioid (E) type cells, which originate from fetal skin and urine; (2) amniotic fluid (AF) type cells which originate from the fetal membranes and trophoblast; and (3) F type cells that originate from fibrous connective tissues and dermal fibroblasts. Only the latter two types (AF type and F type) have been shown to persist in *in vitro* long-term culture. In addition, MSC from the AF (hAF-MSC) have also been described.

Previous studies on hAFC detected cell markers from all three germ layers (von Koskull, 1984; von Koskull et al., 1984; Davydova et al., 2009) proved that a stem cell population is present. Indeed, approximately 1% of the entire hAFC is represented by the stem cell fraction named human amniotic fluid stem cells (hAFSC), that can be clonally expanded by selection with the surface antigen CD117 (De Coppi et al., 2007).

On one hand, hAFSC share many characteristics with MSC such as the positivity for CD73, CD90, CD105, and MHC class I, and lack of MHC class II, CD40, CD80, and CD86 (Moorefield et al., 2011), with low immunogenic profile (Di Trapani et al., 2013) according to gestational age (Di Trapani et al., 2015). On the other hand, hAFSC express pluripotency markers such as SSEA3, SSEA4, NANOG, KLF4 and MYC, TRA1-60 and TRA1-81 (Wolfrum et al., 2010; Moschidou et al., 2013a,b; Zani et al., 2014), although they do not form tumors when injected in mice (Chiavegato et al., 2007; Moschidou et al., 2013b; Bertin et al., 2016). The positivity for OCT-4 is still controversial. Maguire and colleagues reported cytoplasmic and nuclear OCT-4 expression by immunostaining, flow cytometry, clonal analysis, qPCR, and dRNA-seq whole genomic profile (Maguire et al., 2013), on the contrary, a recent study demonstrated the opposite, in particular for mid trimester hAFSC (Vlahova et al., 2019). Nevertheless, hAFSC have been easily reprogrammed not only with DNA-integrating systems (Wolfrum et al., 2010; Bertin et al., 2016) but also without any genetic manipulation by means of the histone deacetylase inhibitor, valproic acid (VPA) (Moschidou et al., 2013a,b).

After CD117 selection hAFSC are either used directly *in vivo* or grown in adhesion, and their natural environment changes from a suspension fluid to a flat surface. Although the antigen surface expression is detected at the first passages (Di Trapani et al., 2013) and is gradually lost during expansion, cell selection makes hAFSC a peculiar population that maintains the ability to differentiate. Indeed, hAFSC can differentiate toward the hematopoietic (Ditadi et al., 2009; Loukogeorgakis et al., 2019), myogenic (Piccoli et al., 2012), and endothelial cells (Schiavo et al., 2015), not only *in vitro* but also *in vivo* and after secondary transplantation. In an elegant work, Xinaris and colleagues performed chimeric kidney organoids with hAFSC (after CD117 selection) and, strikingly, human cells contributed to the formation of glomerular structures, differentiating into podocytes (Xinaris et al., 2016). Neuronal differentiation is still under investigation and even if *in vitro* evidence on protein and function suggests differentiation (De Coppi et al., 2007), it is still less clear *in vivo* (Maraldi et al., 2014). Overall these properties distinguish the stem cell population from the MSC present in AF (see paragraph below).

#### Human Amniotic Fluid Mesenchymal Stromal Cells (hAF-MSC)

Mesenchymal stromal cells from the amniotic fluid (hAF-MSC) are plastic adherent cells defined following the minimal criteria of the consensus paper by Parolini et al. (2008), Spitzhorn et al. (2017). Accordingly, hAF-MSC differentiate toward adipogenic, osteogenic and chondrogenic lineages (Spitzhorn et al., 2017). Of note, the MSC fraction that originates from the hAF is still identified by other surface markers that overlap with some of the already cited proteins expressed by hAFC, such as SSEA4, TRA-1-60, TRA-1-81 (Spitzhorn et al., 2017). hAF-MSC have also been shown to be less prone to senescence with respect to other adult sources of MSC such as bone marrow (Alessio et al., 2018).

### Human Decidua (hD)

The decidua appears in mammals (including humans) with hemochorial placentation and an invasive trophoblast. The human decidua (hD) derives from the endometrium and is therefore of maternal origin. After ovulation, during each menstrual cycle, a reaction called decidualization develops. This process of differentiation involves structural and functional changes in all cells of the endometrium. If menstruation occurs, this tissue is discarded; however, if pregnancy takes place, decidualization continues through the effect of pregnancy hormones (Moffett and Loke, 2006). Three types of hD are distinguished, depending on the spatial relation to the implanting embryo.

#### Human Basal Decidua or Decidua Basalis

Human basal decidua or decidua basalis (hBD) is located between the myometrium and the chorionic plate. The basal decidua is a thin plate of maternal endometrial tissue to which the anchoring villi are attached and which is invaded by extravillous trophoblast. The basal decidua modulates the allocation of maternal-fetal resources. The vessels in the basal decidua supply maternal arterial blood to the intervillous space between the fetal chorionic villi and receive venous blood from the placenta (Benirschke et al., 2012). Importantly, only the invasion of extravillous trophoblast into decidual arteries and veins by endoarterial and endovenous trophoblast cells, respectively, enables the proper vascular flow through the placenta (Huppertz, 2019).

#### Human Capsular Decidua or Decidua Capsularis

Human capsular decidua or decidua capsularis (hCD) is the thin layer of decidua that encapsulates the surface of the chorion laeve towards the uterine lumen. With the growth of the fetus, the capsular decidua is stretched and eventually fuses with the parietal decidua, thereby obliterating the uterine cavity (Benirschke et al., 2012).

#### Human Parietal Decidua or Decidua Parietalis

Human parietal decidua or decidua parietalis (hPD) is the deciduous layer that covers the rest of the uterine cavity. It contains maternal blood vessels and lymphatic vessels to maintain the nutritional and metabolic balance in the maternal compartment. From the third month of pregnancy, the hCD and hPD merge together as a consequence of membrane and fluid increment, as well as of fetal growth and development.

**FIGURE 10:**
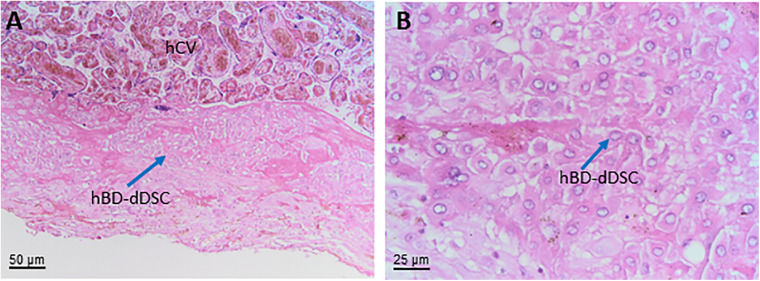
Decidua stromal cells. Histological images of human basal decidua (hBD). Haematoxylin-eosin staining. **(A)** A general overview of the uterine wall containing human basal decidua decidualized stromal cells (hBD-dDSC) representing the maternal component of the human placenta facing the fetal component represented by human chorionic villi (hCV) of the chorion frondosum is appreciable at low magnification (20x). **(B)** At higher magnification (40x) it is possible to appreciate the cell populations present in the human decidua including hBD-dDSC.

Histologically, the hD is composed of decidual stromal cells (DSC, Figure 10), glandular epithelial cells, endothelial cells and numerous leukocytes, with a predominance of large NK CD56 bright cells (which decrease progressively in number as pregnancy progresses), macrophages and T cells, and small proportions of granulocytes and B cells (Yang et al., 2019).

#### Human Decidua Predecidual Stromal Cells (hD-preDSC) and Human Decidualized Decidual Stromal Cells (hD-dDSC)

Decidual stromal cells (DSC), the main cellular component of the hD, exert activities that are thought to play a key role in embryo implantation, trophoblast expansion, and the development of fetal-maternal immune tolerance. These cells originate from fibroblastic precursors located around the vessels and are detected in both the endometrium and decidua. During the luteal phase of the menstrual cycle, under the effect of the ovarian hormones estradiol and progesterone (P4), a predecidual reaction begins around the vessels and spreads through two thirds of the endometrium facing the uterine cavity. Precursors of DSC (preDSC) leave the vessels and differentiate into decidualized cells, which exhibit a rounder shape and secrete prolactin (PRL) and other factors such as insulin-like growth factor-binding protein 1 (IGFBP1) and IL-15, and express dickkopf WNT signaling pathway inhibitor 1 (DKK1), and forkhead box protein O1 (FOXO1). When menstruation occurs, these differentiated cells are discarded; however, if pregnancy takes place, this process of differentiation (decidualization) continues through the effect of pregnancy hormones (Muñoz-Fernández et al., 2018; Vento-Tormo et al., 2018).

There is a great deal of confusion in the terminology related to DSC, but what must be considered is that in both the decidua and endometrium, DSC can be found as undifferentiated and differentiated (decidualized) cells; undifferentiated human DSC are referred to as hD-preDSC (Olivares et al., 1997; Kyurkchiev et al., 2010), and decidualized DSC are called hD-dDSC.

Decidual stromal cells can be obtained from first-trimester decidua (elective termination of pregnancy) (Muñoz-Fernández et al., 2018) or third-trimester decidua (cesarean delivery) (Ringden et al., 2013). The isolation and maintenance of highly purified human DSC lines in culture has made it possible to study the antigen phenotype and activities of these cells. DSC lines exhibit antigen phenotype and functional properties equivalent to those of their corresponding fresh cells. The maternal origin of DSC has been confirmed by microsatellite polymorphism (Ringden et al., 2013; Muñoz-Fernández et al., 2018). In the absence of P4, cAMP, and other decidualizing factors in the culture medium, only hD-preDSC proliferate. In first-trimester decidua, hD-preDSC express CD10 (endometrial stromal cell marker), CD29, CD44, CD54, CD73, CD90, CD105, CD140b, CD146, CD271, alpha SM actin, nestin, OCT3/4, SUS2, podoplanin, STRO-1, and vimentin, and lack CD15, CD19, CD31, CD34, CD45, CD62P, HLA-DR, and cytokeratin (Munoz-Fernandez et al., 2012; Muñoz-Fernández et al., 2018; Vento-Tormo et al., 2018).

Term placental DSC have been less studied than first trimester DSC. In the parietal, capsular and basal decidua, hD-preDSC (hPD-preDSC, hCD-preDSC, hBD-preDSC, respectively) show a phenotype similar to that of the first trimester hD-preDSC (Richards et al., 1995; Oliver et al., 1999; Ringden et al., 2013; Ringden et al., 2018). Likewise, term hD-DSC are decidualized *in vitro* in the presence of P4, as observed in the case of basal (hBD-dDSC) and parietal (hPD-dDSC) human decidualized decidual stromal cells by the secretion or expression of PRL and IGFBP1 (Richards et al., 1995; Oliver et al., 1999). In single cell RNA sequencing studies, term hBD-dDSC have been identified that express the decidualization markers DKK1, PRL, FOXO1, IGFBP1, and IL15 (Pavlicev et al., 2017; Tsang et al., 2017).

Most data on DSC come from studies with hD-preDSC cells from first trimester or term pregnancies, whereby cells are isolated and cultured in a very similar manner: enzymatic digestion (trypsin/EDTA) of decidual tissue followed by *in vitro* culture in DMEM (Richards et al., 1995; Ringden et al., 2013) or Opti-MEM (Muñoz-Fernández et al., 2018). The main methodological difference was that some cultures were in presence of 2%-3% serum (Richards et al., 1995; Muñoz-Fernández et al., 2018) and others in 10% serum (Ringden et al., 2013). The results regarding the antigen phenotype or functions were, however, similar.

#### Human Decidua Mesenchymal Stromal Cells (hDMSC)

Mesenchymal stromal cells that meet the minimal consensus criteria (Dominici et al., 2006; Parolini et al., 2008) can be isolated from first trimester and term hD (Choi et al., 2017). Furthermore, hDMSC are negative for the costimulatory molecules CD40, CD80, CD83 and CD86, and HLA-DR (Huang et al., 2009; Dimitrov et al., 2010; Chen et al., 2015; Abomaray et al., 2016). HLA typing analysis revealed that MSC isolated from the decidua are of maternal origin (In ’t Anker et al., 2004). During the isolation process, the decidual tissue is minced and then digested using collagenase, trypsin or the combination of both. It has been suggested that the collagenase-only protocol is best for hDMSC isolation because of high cell recovery rate (Araújo et al., 2018); but there is currently no study to determine the difference of hDMSC isolated by different methods.

Most studies on hDMSC have been based on cells isolated from the basal decidua (hBD-MSC) (Abomaray et al., 2016), although cells with similar characteristics have been obtained from the parietal decidua in term placenta (hPD-MSC) (In ’t Anker et al., 2004; Kanematsu et al., 2011; Castrechini et al., 2012; Abumaree et al., 2016).

By retroviral transfer of reprogramming factor genes, hBD-MSC have been reprogrammed into pluripotent stem cells that maintain a normal karyotype, share similar characteristics to human embryonic stem cells, and differentiate into the three mesenchymal lineages (Shofuda et al., 2013). hPD-MSC exhibit a typical fibroblast-like morphology and are able to differentiate into adipocytes and chondrocytes but possess a limited potential to differentiate into osteoblasts. Furthermore, these cells were shown to secrete several growth factors and other active molecules which bestow them with functional diversity (Huang et al., 2010; Abumaree et al., 2016), especially concerning their antitumor activity (Bahattab et al., 2019).

In addition, the expression of pericyte-associated antigens such as 3G5, STRO-1, CD146 and αSMA suggested that hPD-MSC may be derived from vascular stem cells (Castrechini et al., 2012). Interestingly, hD-preDSC from first trimester and term decidua also show similar characteristics to those of hDMSC: antigen phenotype, clonogenic capacity, perivascular location, and ability to differentiate into the mesenchymal lineages (Richards et al., 1995; Dimitrov et al., 2010; Castrechini et al., 2012; Muñoz-Fernández et al., 2018). Therefore, it is reasonable to believe that hD-preDSC and the hDMSC described above belong to the same cell population.

## Conclusion

Herein we addressed a few major challenges in the field of perinatal derivatives. First, we provide a thorough description and mapping of the human placenta, including a description of the different cell populations and clarification of where they are located within perinatal tissues. Second, given that the names and abbreviations used to refer to the different cell populations are often misleading and even inappropriate, we propose nomenclature for perinatal tissue and cells. Another important challenge relates to cell isolation and culture/expansion because specific characteristics, such as phenotype, change during culture and can depend, in many cases, on culture conditions. The literature analysis we performed did not allow us to identify specific markers to uniquely identify the cell populations discussed herein. At the current time, this is a *quasi* impossible task due to the heterogeneity of the starting material given by varying isolation and culture protocols. In addition, even though there are a few reports claiming that perinatal cells express markers that are not present in all perinatal regions, a detailed comparison and confirmation by other research groups is needed.

A widespread comparison between isolation protocols, culture conditions, and cell phenotype, remains a critical challenge to be addressed. Therefore, we propose that consortiums active in the field work together to address this challenge. We also propose the adoption of the herein proposed nomenclature based on the precise tissue and cell localization. A defined starting material is the first step towards better data interpretation, study comparison and ultimately, for the establishment of standard cell preparation protocols for clinical use (Brooke et al., 2009; Kabagambe et al., 2017; Lankford et al., 2017; Phinney and Galipeau, 2019; Aghayan et al., 2020).

That having been said, a concerted effort is still required to standardize perinatal cells. Even though the current cell heterogeneity doesn’t seem to be a limiting factor for functionality and therapeutic efficacy, since preclinical studies and initial clinical trials have demonstrated efficacy of different perinatal cells. Rather, standardizing cells and deciphering cell heterogeneity is of utmost importance to potentially fine-tune cells for specific therapeutic applications and to select cells that will provide an optimal response to the disease.

## Author Contributions

AS, OP wrote the Introduction. FA, MB, RDP wrote section “Development of the Early Placenta”. RDP, BH wrote section “Structure of the Early Placenta”. RDP, FA wrote section “Structure of the Term Placenta”. All authors contributed to section “Cells Isolated from Term Placenta”. ILO wrote section “Blood Vessels of the Human Term Placenta and Umbilical Cord”. ILO, BH, RDP, MB, OS, SS, AS prepared and revised the Figures. AS and OP coordinated the work and compiled the manuscript. All authors contributed to revising and editing the manuscript and approved the manuscript.

## Conflict of Interest

The authors declare that the research was conducted in the absence of any commercial or financial relationships that could be construed as a potential conflict of interest.

## References

[B1] Abbaszadeh H. Ghorbani F. Derakhshani M. Movassaghpour A. A. Yousefi M. Talebi M. (2020). Regenerative potential of Wharton’s jelly-derived mesenchymal stem cells: a new horizon of stem cell therapy. *J. Cell. Physiol.* 235 9230–9240. 10.1002/jcp.29810 32557631

[B2] Abomaray F. M. Al Jumah M. A. Alsaad K. O. Jawdat D. Al Khaldi A. AlAskar A. S. (2016). Phenotypic and functional characterization of mesenchymal stem/multipotent stromal cells from decidua basalis of human term placenta. *Stem Cells Int.* 2016:5184601. 10.1155/2016/5184601 PMC4764756 27087815

[B3] Abomaray F. M. Al Jumah M. A. Kalionis B. AlAskar A. S. Al Harthy S. (2015). Human chorionic villous mesenchymal stem cells modify the functions of human dendritic cells, and induce an anti-inflammatory phenotype in CD1+ dendritic cells. *Stem Cell Rev. Rep.* 11 423–441. 10.1007/s12015-014-9562-8 25287760

[B4] Abumaree M. H. Abomaray F. M. Alshehri N. A. Almutairi A. AlAskar A. S. Al Jumah M. A. (2016). Phenotypic and Functional Characterization of Mesenchymal Stem/Multipotent Stromal Cells From Decidua Parietalis of Human Term Placenta. *Reprod Sci* 23 1193–1207. 10.1177/1933719116632924 26902429

[B5] Aghayan H. R. Payab M. Mohamadi-Jahani F. Aghayan S. S. Larijani B. Arjmand B. (2020). *GMP-Compliant Production of Human Placenta-Derived Mesenchymal Stem Cells.* (New York, NY: Springer), 1–13. 10.1007/7651_2020_282 32504292

[B6] Alcaraz A. Mrowiec A. Insausti C. L. García-Vizcaíno E. M. Ruiz-Canada C. López-Martínez M. C. (2013). Autocrine TGF-β induces epithelial to mesenchymal transition in human amniotic epithelial cells. *Cell Transplant.* 22 1351–1367. 10.3727/096368912x657387 23031712

[B7] Alessio N. Pipino C. Mandatori D. Ferone A. Marchiso M. Melone M. A. B. (2018). Mesenchymal stromal cells from amniotic fluid are less prone to senescence compared to those obtained from bone marrow: an *in vitro* study. *J. Cell. Physiol.* 233 8996–9006. 10.1002/jcp.26845 29904927

[B8] Alviano F. Fossati V. Marchionni C. Arpinati M. Bonsi L. Franchina M. (2007). Term amniotic membrane is a high throughput source for multipotent mesenchymal stem cells with the ability to differentiate into endothelial cells *in vitro*. *BMC Dev. Biol.* 7:11. 10.1186/1471-213X-7-11 17313666 PMC1810523

[B9] Aplin J. D. (2010). Developmental cell biology of human villous trophoblast: current research problems. *Int. J. Dev. Biol.* 54 323–329. 10.1387/ijdb.082759ja 19876840

[B10] Araújo A. B. Furlan J. M. Salton G. D. Schmalfuss T. Röhsig L. M. Silla L. M. R. (2018). Isolation of human mesenchymal stem cells from amnion, chorion, placental decidua and umbilical cord: comparison of four enzymatic protocols. *Biotechnol. Lett.* 40 989–998. 10.1007/s10529-018-2546-z 29619744

[B11] Avolio E. Alvino V. V. Ghorbel M. T. Campagnolo P. (2017). Perivascular cells and tissue engineering: current applications and untapped potential. *Pharmacol. Ther.* 171 83–92. 10.1016/j.pharmthera.2016.11.002 27889329 PMC5345698

[B12] Baczyk D. Drewlo S. Proctor L. Dunk C. Lye S. Kingdom J. (2009). Glial cell missing-1 transcription factor is required for the differentiation of the human trophoblast. *Cell Death Differ.* 16 719–727. 10.1038/cdd.2009.1 19219068

[B13] Bahattab E. Khatlani T. Abomaray F. M. Messaoudi S. A. Abumaree M. H. (2019). Cancer conditioned medium modulates functional and phenotypic properties of human decidua parietalis mesenchymal stem/stromal cells. *Tissue Eng. Regen. Med.* 16 615–630. 10.1007/s13770-019-00207-w 31824824 PMC6879693

[B14] Bailo M. Soncini M. Vertua E. Signoroni P. B. Sanzone S. Lombardi G. (2004). Engraftment potential of human amnion and chorion cells derived from term placenta. *Transplantation* 78 1439–1448. 10.1097/01.tp.0000144606.84234.49 15599307

[B15] Banas R. A. Trumpower C. Bentlejewski C. Marshall V. Sing G. Zeevi A. (2008). Immunogenicity and immunomodulatory effects of amnion-derived multipotent progenitor cells. *Hum. Immunol.* 69 321–328. 10.1016/j.humimm.2008.04.007 18571002

[B16] Banerjee A. Lindenmair A. Hennerbichler S. Steindorf P. Steinborn R. Kozlov A. V. (2018). Cellular and site-specific mitochondrial characterization of vital human amniotic membrane. *Cell Transplant.* 27 3–11. 10.1177/0963689717735332 29562784 PMC6434485

[B17] Banerjee A. Weidinger A. Hofer M. Steinborn R. Lindenmair A. Hennerbichler-Lugscheider S. (2015). Different metabolic activity in placental and reflected regions of the human amniotic membrane. *Placenta* 36 1329–1332. 10.1016/j.placenta.2015.08.015 26386652

[B18] Benirschke K. Kaufmann P. Baergen R. N. (2006). *Pathology of the Human Placenta*, 5th Edn New York, NY: Springer-Verlag.

[B19] Benirschke K. Burton G. J. Baergen R. N. (eds) (2012). *Pathology of the Human Placenta.* New York, NY: Springer.

[B20] Bertin E. Piccoli M. Franzin C. Spiro G. Donà S. Dedja A. (2016). First steps to define murine amniotic fluid stem cell microenvironment. *Sci. Rep.* 6:37080. 10.1038/srep37080 PMC5109045 27845396

[B21] Bilic G. Zeisberger S. M. Mallik A. S. Zimmermann R. Zisch A. H. (2008). Comparative characterization of cultured human term amnion epithelial and mesenchymal stromal cells for application in cell therapy. *Cell Transplant.* 17 955–968. 10.3727/096368908786576507 19069637

[B22] Blaschitz A. Gauster M. Fuchs D. Lang I. Maschke P. Ulrich D. (2011). Vascular endothelial expression of indoleamine 2,3-dioxygenase 1 forms a positive gradient towards the feto-maternal interface. *PLoS One* 6:e21774. 10.1371/journal.pone.0021774 21755000 PMC3130744

[B23] Bollini S. Silini A. R. Banerjee A. Wolbank S. Balbi C. Parolini O. (2018). Cardiac restoration stemming from the placenta tree: insights from fetal and perinatal cell biology. *Front. Physiol.* 9:385. 10.3389/fphys.2018.00385 29695981 PMC5904405

[B24] Brooke G. Rossetti T. Pelekanos R. Ilic N. Murray P. Hancock S. (2009). Manufacturing of human placenta-derived mesenchymal stem cells for clinical trials. *Br. J. Haematol.* 144 571–579. 19077161 10.1111/j.1365-2141.2008.07492.x

[B25] Burton G. J. Fowden A. L. (2015). The placenta: a multifaceted, transient organ. *Philos. Trans. R. Soc. Lond. B Biol. Sci.* 370:20140066. 10.1098/rstb.2014.0066 25602070 PMC4305167

[B26] Can A. Balci D. (2011). Isolation, culture, and characterization of human umbilical cord stroma-derived mesenchymal stem cells. *Methods Mol. Biol.* 698 51–62. 10.1007/978-1-60761-999-4_5 21431510

[B27] Carlson B. M. (2013). *Human Embryology and Developmental Biology*, 5th Edn Philadelphia, PA: Elsevier Saunders.

[B28] Caruso M. Evangelista M. Parolini O. (2012). Human term placental cells: phenotype, properties and new avenues in regenerative medicine. *Int. J. Mol. Cell. Med.* 1 64–74. 24551761 PMC3920494

[B29] Casanello P. Schneider D. Herrera E. Uauy R. Krause B. (2014). Endothelial heterogeneity in the umbilico-placental unit: DNA methylation as an innuendo of epigenetic diversity. *Front. Pharmacol.* 5:49. 10.3389/fphar.2014.00049 24723887 PMC3973902

[B30] Casey M. L. MacDonald P. C. (1997). Keratinocyte growth factor expression in the mesenchymal cells of human amnion. *J. Clin. Endocrinol. Metab.* 82 3319–3323. 10.1210/jc.82.10.3319 9329361

[B31] Castrechini N. M. Murthi P. Gude N. M. Erwich J. J. Gronthos S. Zannettino A. (2010). Mesenchymal stem cells in human placental chorionic villi reside in a vascular Niche. *Placenta* 31 203–212. 10.1016/j.placenta.2009.12.006 20060164

[B32] Castrechini N. M. Murthi P. Qin S. Kusuma G. D. Wilton L. Abumaree M. (2012). Decidua parietalis-derived mesenchymal stromal cells reside in a vascular niche within the choriodecidua. *Reprod. Sci.* 19 1302–1314. 10.1177/1933719112450334 22886285

[B33] Cauffman G. Liebaers I. Van Steirteghem A. Van de Velde H. (2006). POU5F1 isoforms show different expression patterns in human embryonic stem cells and preimplantation embryos. *Stem Cells* 24 2685–2691. 10.1634/stemcells.2005-0611 16916925

[B34] Centurione L. Passaretta F. Centurione M. A. Munari S. Vertua E. Silini A. (2018). Mapping of the human placenta: experimental evidence of amniotic epithelial cell heterogeneity. *Cell Transplant.* 27 12–22. 10.1177/0963689717725078 29562779 PMC6434477

[B35] Challier J. C. Kacemi A. Olive G. (1995). Mixed culture of pericytes and endothelial cells from fetal microvessels of the human placenta. *Cell. Mol. Biol.* 41 233–241. 7787733

[B36] Chen G. Yue A. Ruan Z. Yin Y. Wang R. Ren Y. (2015). Comparison of biological characteristics of mesenchymal stem cells derived from maternal-origin placenta and Wharton’s jelly. *Stem Cell Res. Ther.* 6:228. 10.1186/s13287-015-0219-6 PMC4660673 26607396

[B37] Chen L. Merkhan M. M. Forsyth N. R. Wu P. (2019). Chorionic and amniotic membrane-derived stem cells have distinct, and gestational diabetes mellitus independent, proliferative, differentiation, and immunomodulatory capacities. *Stem Cell Res.* 40:101537. 10.1016/j.scr.2019.101537 31422237

[B38] Chiavegato A. Bollini S. Pozzobon M. Callegari A. Gasparotto L. Taiani J. (2007). Human amniotic fluid-derived stem cells are rejected after transplantation in the myocardium of normal, ischemic, immuno-suppressed or immuno-deficient rat. *J. Mol. Cell Cardiol.* 42 746–759. 10.1016/j.yjmcc.2006.12.008 17300799

[B39] Choi Y. S. Park Y. B. Ha C. W. Kim J. A. Heo J. C. Han W. J. (2017). Different characteristics of mesenchymal stem cells isolated from different layers of full term placenta. *PLoS One* 12:e0172642. 10.1371/journal.pone.0172642 28225815 PMC5321410

[B40] Cirman T. Beltram M. Schollmayer P. Rožman P. Kreft M. E. (2014). Amniotic membrane properties and current practice of amniotic membrane use in ophthalmology in Slovenia. *Cell Tissue Bank* 15 177–192. 10.1007/s10561-013-9417-6 24352631

[B41] Conconi M. T. Rosa D. L. Mara T. Calore C. Pier P. P. (2011). Phenotype and differentiation potential of stromal populations obtained from various zones of human umbilical cord: an overview. *Open Tissue Eng. Regen. Med. J.* 4 6–20. 10.2174/1875043501104010006

[B42] Corrao S. La Rocca G. Lo Iacono M. Corsello T. Farina F. Anzalone R. (2013). Umbilical cord revisited: from Wharton’s jelly myofibroblasts to mesenchymal stem cells. *Histol. Histopathol.* 28 1235–1244. 23595555 10.14670/HH-28.1235

[B43] Couto P. S. Bersenev A. Verter F. (2017). The first decade of advanced cell therapy clinical trials using perinatal cells. (2005-2015). *Regen. Med.* 12 953–968. 10.2217/rme-2017-0066 29139329

[B44] Davies J. E. Walker J. T. Keating A. (2017). Concise review: Wharton’s Jelly: the rich, but enigmatic, source of mesenchymal stromal cells. *Stem Cells Transl. Med.* 6 1620–1630. 10.1002/sctm.16-0492 28488282 PMC5689772

[B45] Davydova D. A. Vorotelyak E. A. Smirnova Y. A. Zinovieva R. D. Romanov Y. A. Kabaeva N. V. (2009). Cell phenotypes in human amniotic fluid. *Acta Nat.* 1 98–103. PMC3347518 22649611

[B46] De Coppi P. Bartsch G. Jr. Siddiqui M. M. Xu T. Santos C. C. Perin L. (2007). Isolation of amniotic stem cell lines with potential for therapy. *Nat. Biotechnol.* 25 100–106. 10.1038/nbt1274 17206138

[B47] De Coppi P. Bartsch G. Atala A. (2009). “18 - Amniotic fluid and placental stem cells as a source for urological regenerative medicine,” in *Biomaterials and Tissue Engineering in Urology*, eds Denstedt J. Atala A. (Sawston: Woodhead Publishing), 378–394. 10.1533/9781845696375.3.378

[B48] Demir R. Seval Y. Huppertz B. (2007). Vasculogenesis and angiogenesis in the early human placenta. *Acta Histochem.* 109 257–265. 10.1016/j.acthis.2007.02.008 17574656

[B49] Deuse T. Stubbendorff M. Phillips N. Kay M. A. Eiermann T. Phan T. T. (2011). Immunogenicity and immunomodulatory properties of umbilical cord lining mesenchymal stem cells. *Cell Transplant.* 20 655–667. 10.3727/096368910x536473 21054940

[B50] Di Trapani M. Bassi G. Fontana E. Giacomello L. Pozzobon M. Guillot P. V. (2015). Immune regulatory properties of CD117(pos). amniotic fluid stem cells vary according to gestational age. *Stem Cells Dev.* 24 132–143. 10.1089/scd.2014.0234 25072397 PMC4273183

[B51] Di Trapani M. Bassi G. Ricciardi M. Fontana E. Bifari F. Pacelli L. (2013). Comparative study of immune regulatory properties of stem cells derived from different tissues. *Stem Cells Dev.* 22 2990–3002. 10.1089/scd.2013.0204 23819720 PMC3840473

[B52] Díaz-Prado S. Muñios-López E. Hermida-Gómez T. Rendal-Vázquez M. E. Fuentes-Boquete I. de Toro F. J. (2010). Multilineage differentiation potential of cells isolated from the human amniotic membrane. *J. Cell. Biochem.* 111 846–857. 10.1002/jcb.22769 20665539

[B53] Dimitrov R. Kyurkchiev D. Timeva T. Yunakova M. Stamenova M. Shterev A. (2010). First-trimester human decidua contains a population of mesenchymal stem cells. *Fertil. Steril.* 93 210–219. 10.1016/j.fertnstert.2008.09.061 19006798

[B54] Ditadi A. de Coppi P. Picone O. Gautreau L. Smati R. Six E. (2009). Human and murine amniotic fluid c-Kit+Lin- cells display hematopoietic activity. *Blood* 113 3953–3960. 10.1182/blood-2008-10-182105 19221036

[B55] Dobreva M. P. Pereira P. N. Deprest J. Zwijsen A. (2010). On the origin of amniotic stem cells: of mice and men. *Int. J. Dev. Biol.* 54 761–777. 10.1387/ijdb.092935md 20446274

[B56] Dominici M. Le Blanc K. Mueller I. Slaper-Cortenbach I. Marini F. Krause D. (2006). Minimal criteria for defining multipotent mesenchymal stromal cells. The International Society for Cellular Therapy position statement. *Cytotherapy* 8 315–317. 10.1080/14653240600855905 16923606

[B57] Dong C. Beltcheva M. Gontarz P. Zhang B. Popli P. Fischer L. A. (2020). Derivation of trophoblast stem cells from naïve human pluripotent stem cells. *eLife* 9:e52504. 10.7554/eLife.52504 PMC7062471 32048992

[B58] Dye J. F. Jablenska R. Donnelly J. L. Lawrence L. Leach L. Firth J. A. (2001). Phenotype of the endothelium in the human term placenta. *Placenta* 22 32–43. 10.1053/plac.2000.0579 11162350

[B59] Enders A. C. Carter A. M. (2004). What can comparative studies of placental structure tell us?–A review. *Placenta* 25(Suppl. A) S3–S9. 15033300 10.1016/j.placenta.2004.01.011

[B60] Escudero C. Casanello P. Sobrevia L. (2008). Human equilibrative nucleoside transporters 1 and 2 may be differentially modulated by A2B adenosine receptors in placenta microvascular endothelial cells from pre-eclampsia. *Placenta* 29 816–825. 10.1016/j.placenta.2008.06.014 18703227

[B61] Evron A. Goldman S. Shalev E. (2011). Human amniotic epithelial cells cultured in substitute serum medium maintain their stem cell characteristics for up to four passages. *Int. J. Stem Cells* 4 123–132. 10.15283/ijsc.2011.4.2.123 24298345 PMC3840962

[B62] Fauza D. (2004). Amniotic fluid and placental stem cells. *Best Pract. Res. Clin. Obstet. Gynaecol.* 18 877–891. 10.1016/j.bpobgyn.2004.07.001 15582544

[B63] Fock V. Plessl K. Fuchs R. Dekan S. Milla S. K. Haider S. (2015). Trophoblast subtype-specific EGFR/ERBB4 expression correlates with cell cycle progression and hyperplasia in complete hydatidiform moles. *Hum. Reprod.* 30 789–799. 10.1093/humrep/dev027 25740878

[B64] Fong C. Y. Chak L. L. Biswas A. Tan J. H. Gauthaman K. Chan W. K. (2011). Human Wharton’s jelly stem cells have unique transcriptome profiles compared to human embryonic stem cells and other mesenchymal stem cells. *Stem Cell Rev. Rep.* 7 1–16. 10.1007/s12015-010-9166-x 20602182

[B65] Fukuchi Y. Nakajima H. Sugiyama D. Hirose I. Kitamura T. Tsuji K. (2004). Human placenta-derived cells have mesenchymal stem/progenitor cell potential. *Stem Cells* 22 649–658. 10.1634/stemcells.22-5-649 15342929

[B66] Gamage T. K. Chamley L. W. James J. L. (2016). Stem cell insights into human trophoblast lineage differentiation. *Hum. Reprod. Update* 23 77–103. 10.1093/humupd/dmw026 27591247

[B67] Gao K. He S. Kumar P. Farmer D. Zhou J. Wang A. (2020). Clonal isolation of endothelial colony-forming cells from early gestation chorionic villi of human placenta for fetal tissue regeneration. *World J. Stem Cells* 12 123–138. 10.4252/wjsc.v12.i2.123 32184937 PMC7062038

[B68] Gaus G. Funayama H. Huppertz B. Kaufmann P. Frank H. G. (1997). Parent cells for trophoblast hybridization I: isolation of extravillous trophoblast cells from human term chorion laeve. *Placenta* 18 181–190. 10.1016/s0143-4004(97)80088-49089780

[B69] Genbacev O. Larocque N. Ona K. Prakobphol A. Garrido-Gomez T. Kapidzic M. (2016). Integrin α4-positive human trophoblast progenitors: functional characterization and transcriptional regulation. *Hum. Reprod.* 31 1300–1314. 10.1093/humrep/dew077 27083540 PMC4871193

[B70] Gooi H. C. Feizi T. Kapadia A. Knowles B. B. Solter D. Evans M. J. (1981). Stage-specific embryonic antigen involves αl→ 3 fucosylated type 2 blood group chains. *Nature* 292 156–158. 10.1038/292156a0 6165896

[B71] Guan Y. T. Xie Y. Li D. S. Zhu Y. Y. Zhang X. L. Feng Y. L. (2019). Comparison of biological characteristics of mesenchymal stem cells derived from the human umbilical cord and decidua parietalis. *Mol. Med. Rep.* 20 633–639. 31180542 10.3892/mmr.2019.10286 PMC6579987

[B72] Haider S. Meinhardt G. Saleh L. Kunihs V. Gamperl M. Kaindl U. (2018). Self-renewing trophoblast organoids recapitulate the developmental program of the early human placenta. *Stem Cell Rep.* 11 537–551. 10.1016/j.stemcr.2018.07.004 30078556 PMC6092984

[B73] Hannah M. J. Williams R. Kaur J. Hewlett L. J. Cutler D. F. (2002). Biogenesis of Weibel-Palade bodies. *Semin. Cell Dev. Biol.* 13 313–324. 10.1016/s1084-9521(02)00061-7 12243731

[B74] Hartmann I. Hollweck T. Haffner S. Krebs M. Meiser B. Reichart B. (2010). Umbilical cord tissue-derived mesenchymal stem cells grow best under GMP-compliant culture conditions and maintain their phenotypic and functional properties. *J. Immunol. Methods* 363 80–89. 10.1016/j.jim.2010.10.008 21035451

[B75] Holthöfer H. Virtanen I. Kariniemi A. L. Hormia M. Linder E. Miettinen A. (1982). Ulex europaeus I lectin as a marker for vascular endothelium in human tissues. *Lab. Invest.* 47 60–66. 6177923

[B76] Horii M. Li Y. Wakeland A. K. Pizzo D. P. Nelson K. K. Sabatini K. (2016). Human pluripotent stem cells as a model of trophoblast differentiation in both normal development and disease. *Proc. Natl. Acad. Sci. U.S.A.* 113 E3882. 10.1073/pnas.1604747113 PMC4941448 27325764

[B77] Hormia M. Lehto V. P. Virtanen I. (1983). Identification of UEA I-binding surface glycoproteins of cultured human endothelial cells. *Cell Biol. Int. Rep.* 7 467–475. 10.1016/0309-1651(83)90136-4 6883521

[B78] Hou M. Han J. Li G. Jiang J. Emani S. Taglauer E. S. (2020). Multipotency of mouse trophoblast stem cells. *Stem Cell Res. Ther.* 11:55. 10.1186/s13287-020-1567-4 PMC7020558 32054514

[B79] Huang L. Wong Y. P. Gu H. Cai Y. J. Ho Y. Wang C. C. (2011). Stem cell-like properties of human umbilical cord lining epithelial cells and the potential for epidermal reconstitution. *Cytotherapy* 13 145–155. 10.3109/14653249.2010.509578 20735166

[B80] Huang Q. Yang Y. Luo C. Wen Y. Liu R. Li S. (2019). An efficient protocol to generate placental chorionic plate-derived mesenchymal stem cells with superior proliferative and immunomodulatory properties. *Stem Cell Res. Ther.* 10:301. 10.1186/s13287-019-1405-8 PMC6796371 31623677

[B81] Huang Y. C. Yang Z. M. Chen X. H. Tan M. Y. Wang J. Li X. Q. (2009). Isolation of mesenchymal stem cells from human placental decidua basalis and resistance to hypoxia and serum deprivation. *Stem Cell Rev. Rep.* 5 247–255. 10.1007/s12015-009-9069-x 19590988

[B82] Huang Y. C. Yang Z. M. Jiang N. G. Chen X. H. Li X. Q. Tan M. Y. (2010). Characterization of MSCs from human placental decidua basalis in hypoxia and serum deprivation. *Cell Biol. Int.* 34 237–243. 10.1042/cbi20090044 19947920

[B83] Huppertz B. (2008). The anatomy of the normal placenta. *J. Clin. Pathol.* 61 1296–1302. 10.1136/jcp.2008.055277 18755720

[B84] Huppertz B. (2019). Traditional and new routes of trophoblast invasion and their implications for pregnancy diseases. *Int. J. Mol. Sci.* 21:289. 10.3390/ijms21010289 31906245 PMC6981830

[B85] Huppertz B. Frank H. G. Kingdom J. C. Reister F. Kaufmann P. (1998). Villous cytotrophoblast regulation of the syncytial apoptotic cascade in the human placenta. *Histochem. Cell Biol.* 110 495–508. 10.1007/s004180050311 9826129

[B86] Huppertz B. Ghosh D. Sengupta J. (2014). An integrative view on the physiology of human early placental villi. *Prog. Biophys. Mol. Biol.* 114 33–48. 10.1016/j.pbiomolbio.2013.11.007 24291663

[B87] Huppertz B. Peeters L. L. (2005). Vascular biology in implantation and placentation. *Angiogenesis* 8 157–167. 10.1007/s10456-005-9007-8 16211358

[B88] Igura K. Zhang X. Takahashi K. Mitsuru A. Yamaguchi S. Takashi T. A. (2004). Isolation and characterization of mesenchymal progenitor cells from chorionic villi of human placenta. *Cytotherapy* 6 543–553. 10.1080/14653240410005366-1 15770794

[B89] In ’t Anker P. S. Scherjon S. A. Kleijburg-van der Keur C. de Groot-Swings G. M. Claas F. H. Fibbe W. E. (2004). Isolation of mesenchymal stem cells of fetal or maternal origin from human placenta. *Stem Cells* 22 1338–1345. 10.1634/stemcells.2004-0058 15579651

[B90] Insausti C. L. Blanquer M. Bleda P. Iniesta P. Majado M. J. Moraleda J. M. (2010). The amniotic membrane as a source of stem cells. *Histol. Histopathol.* 25 91–98. 19924645 10.14670/HH-25.91

[B91] Izumi M. Pazin B. J. Minervini C. F. Gerlach J. Ross M. A. Stolz D. B. (2009). Quantitative comparison of stem cell marker-positive cells in fetal and term human amnion. *J. Reprod. Immunol.* 81 39–43. 10.1016/j.jri.2009.02.007 19501410

[B92] James J. L. Carter A. M. Chamley L. W. (2012). Human placentation from nidation to 5 weeks of gestation. Part I: What do we know about formative placental development following implantation? *Placenta* 33 327–334. 10.1016/j.placenta.2012.01.020 22374510

[B93] Jerman U. D. Veraniè P. Kreft M. E. (2014). Amniotic membrane scaffolds enable the development of tissue-engineered urothelium with molecular and ultrastructural properties comparable to that of native urothelium. *Tissue Eng. Part C Methods* 20 317–327. 10.1089/ten.tec.2013.0298 23947657 PMC3968885

[B94] Jinga V. V. Gafencu A. Antohe F. Constantinescu E. Heltianu C. Raicu M. (2000). Establishment of a pure vascular endothelial cell line from human placenta. *Placenta* 21 325–336. 10.1053/plac.1999.0492 10833367

[B95] Joerger-Messerli M. S. Marx C. Oppliger B. Mueller M. Surbek D. V. Schoeberlein A. (2016). Mesenchymal stem cells from Wharton’s Jelly and amniotic fluid. *Best Pract. Res. Clin. Obstetr. Gynaecol.* 31 30–44. 10.1016/j.bpobgyn.2015.07.006 26482184

[B96] Joo J. E. Hiden U. Lassance L. Gordon L. Martino D. J. Desoye G. (2013). Variable promoter methylation contributes to differential expression of key genes in human placenta-derived venous and arterial endothelial cells. *BMC Genomics* 14:475. 10.1186/1471-2164-14-475 23855827 PMC3729658

[B97] Kabagambe S. Keller B. Becker J. Goodman L. Pivetti C. Lankford L. (2017). Placental mesenchymal stromal cells seeded on clinical grade extracellular matrix improve ambulation in ovine myelomeningocele. *J. Pediatr. Surg.* 10.1016/j.jpedsurg.2017.10.032 [Epub ahead of print]. 29122293

[B98] Kacémi A. Galtier M. Espié M. J. Challier J. C. (1997). Isolation of villous microvessels from the human placenta. *C. R. Acad. Sci. III* 320 171–177. 10.1016/s0764-4469(97)85009-3 9181123

[B99] Kanematsu D. Shofuda T. Yamamoto A. Ban C. Ueda T. Yamasaki M. (2011). Isolation and cellular properties of mesenchymal cells derived from the decidua of human term placenta. *Differentiation* 82 77–88. 10.1016/j.diff.2011.05.010 21684674

[B100] Kang J. W. Koo H. C. Hwang S. Y. Kang S. K. Ra J. C. Lee M. H. (2012). Immunomodulatory effects of human amniotic membrane-derived mesenchymal stem cells. *J. Vet. Sci.* 13 23–31. 10.4142/jvs.2012.13.1.23 22437532 PMC3317453

[B101] Karahuseyinoglu S. Cinar O. Kilic E. Kara F. Akay G. G. Demiralp D. O. (2007). Biology of stem cells in human umbilical cord stroma: in situ and *in vitro* surveys. *Stem Cells* 25 319–331. 10.1634/stemcells.2006-0286 17053211

[B102] Karlsson H. Erkers T. Nava S. Ruhm S. Westgren M. Ringdén O. (2012). Stromal cells from term fetal membrane are highly suppressive in allogeneic settings *in vitro*. *Clin. Exp. Immunol.* 167 543–555. 10.1111/j.1365-2249.2011.04540.x 22288598 PMC3374287

[B103] Kaufmann P. (1981). “Entwicklung der Plazenta,” in *Die Plazenta des Menschen*, eds. Becker V. Schiebler T. H. Kubli F. (Stuttgart: Thieme), 13–50.

[B104] Kaufmann P. Bruns U. Leiser R. Luckhardt M. Winterhager E. (1985). The fetal vascularisation of term human placental villi. II. Intermediate and terminal villi. *Anat. Embryol.* 173 203–214. 10.1007/bf00316301 4083522

[B105] Kim J. Kang H. M. Kim H. Kim M. R. Kwon H. C. Gye M. C. (2007). *Ex vivo* characteristics of human amniotic membrane-derived stem cells. *Cloning Stem Cells* 9 581–594. 10.1089/clo.2007.0027 18154518

[B106] Kim M. J. Shin K. S. Jeon J. H. Lee D. R. Shim S. H. Kim J. K. (2011). Human chorionic-plate-derived mesenchymal stem cells and Wharton’s jelly-derived mesenchymal stem cells: a comparative analysis of their potential as placenta-derived stem cells. *Cell Tissue Res.* 346 53–64. 10.1007/s00441-011-1249-8 21987220

[B107] King A. Boocock C. Sharkey A. M. Gardner L. Beretta A. Siccardi A. G. (1996). Evidence for the expression of HLAA-C class I mRNA and protein by human first trimester trophoblast. *J. Immunol.* 156 2068–2076. 8690894

[B108] Kita K. Gauglitz G. G. Phan T. T. Herndon D. N. Jeschke M. G. (2010). Isolation and characterization of mesenchymal stem cells from the sub-amniotic human umbilical cord lining membrane. *Stem Cells Dev.* 19 491–502. 10.1089/scd.2009.0192 19635009

[B109] Knöfler M. Haider S. Saleh L. Pollheimer J. Gamage J. B. James J. (2019). Human placenta and trophoblast development: key molecular mechanisms and model systems. *Cell. Mol Life Sci.* 76 3479–3496. 10.1007/s00018-019-03104-6 31049600 PMC6697717

[B110] Konig J. Huppertz B. Desoye G. Parolini O. Frohlich J. D. Weiss G. (2012). Amnion-derived mesenchymal stromal cells show angiogenic properties but resist differentiation into mature endothelial cells. *Stem Cells Dev.* 21 1309–1320. 10.1089/scd.2011.0223 21762016

[B111] Konig J. Weiss G. Rossi D. Wankhammer K. Reinisch A. Kinzer M. (2015). Placental mesenchymal stromal cells derived from blood vessels or avascular tissues: What is the better choice to support endothelial cell function?. *Stem Cells Dev.* 24 115–131. 10.1089/scd.2014.0115 25244528 PMC4273191

[B112] Kwon A. Kim Y. Kim M. Kim J. Choi H. Jekarl D. W. (2016). Tissue-specific differentiation potency of mesenchymal stromal cells from perinatal tissues. *Sci. Rep.* 6:23544. 10.1038/srep23544 PMC4820697 27045658

[B113] Kyurkchiev S. Shterev A. Dimitrov R. (2010). Assessment of presence and characteristics of multipotent stromal cells in human endometrium and decidua. *Reprod. Biomed. Online* 20 305–313. 10.1016/j.rbmo.2009.12.011 20117049

[B114] La Rocca G. Anzalone R. Corrao S. Magno F. Loria T. Giannuzzi P. (2009). Isolation and characterization of Oct-4+/HLA-G+ mesenchymal stem cells from human umbilical cord matrix: differentiation potential and detection of new markers. *Histochem. Cell Biol.* 131 267–282. 10.1007/s00418-008-0519-3 18836737

[B115] Lang I. Hahn T. Dohr G. Skofitsch G. Desoye G. (1994). Heterogeneous histochemical reaction pattern of the lectin Bandeiraea. (Griffonia). simplicifolia with blood vessels of human full-term placenta. *Cell Tissue Res.* 278 433–438. 10.1007/s004410050233 7850854

[B116] Lang I. Hartmann M. Blaschitz A. Dohr G. Skofitsch G. Desoye G. (1993). Immunohistochemical evidence for the heterogeneity of maternal and fetal vascular endothelial cells in human full-term placenta. *Cell Tissue Res.* 274 211–218. 10.1007/bf00318740 7505718

[B117] Lang I. Pabst M. A. Hiden U. Blaschitz A. Dohr G. Hahn T. (2003). Heterogeneity of microvascular endothelial cells isolated from human term placenta and macrovascular umbilical vein endothelial cells. *Eur. J. Cell Biol.* 82 163–173. 10.1078/0171-9335-00306 12751902

[B118] Lang I. Schweizer A. Hiden U. Hagendorfer G. Bilban M. Pabst M. A. (2008). Human fetal placental endothelial cells have a mature arterial and a juvenile venous phenotype with adipogenic and osteogenic differentiation potential. *Differentiation* 76 1031–1043. 10.1111/j.1432-0436.2008.00302.x 18673379

[B119] Lankford L. Chen Y. J. Saenz Z. Kumar P. Long C. Farmer D. (2017). Manufacture and preparation of human placenta-derived mesenchymal stromal cells for local tissue delivery. *Cytotherapy* 19 680–688. 10.1016/j.jcyt.2017.03.003 28438482

[B120] Lankford L. Selby T. Becker J. Ryzhuk V. Long C. Farmer D. (2015). Early gestation chorionic villi-derived stromal cells for fetal tissue engineering. *World J. Stem Cells* 7 195–207. 10.4252/wjsc.v7.i1.195 25621120 PMC4300931

[B121] Leach L. Bhasin Y. Clark P. Firth J. A. (1994). Isolation of endothelial cells from human term placental villi using immunomagnetic beads. *Placenta* 15 355–364. 10.1016/0143-4004(94)90003-5 7937593

[B122] Lee C. Q. E. Gardner L. Turco M. Zhao N. Murray M. J. Coleman N. (2016). What is trophoblast? A combination of criteria define human first-trimester trophoblast. *Stem cell Rep.* 6 257–272. 10.1016/j.stemcr.2016.01.006 26862703 PMC4750161

[B123] Lee J. Kim H. K. Rho J. Y. Han Y. M. Kim J. (2006). The human OCT-4 isoforms differ in their ability to confer self-renewal. *J. Biol. Chem.* 281 33554–33565. 10.1074/jbc.m603937200 16951404

[B124] Lee O. K. Kuo T. K. Chen W. M. Lee K. D. Hsieh S. L. Chen T. H. (2004). Isolation of multipotent mesenchymal stem cells from umbilical cord blood. *Blood* 103 1669–1675. 10.1182/blood-2003-05-1670 14576065

[B125] Lemke A. Castillo-Sánchez J. C. Prodinger F. Ceranic A. Hennerbichler-Lugscheider S. Pérez-Gil J. (2017). Human amniotic membrane as newly identified source of amniotic fluid pulmonary surfactant. *Sci. Rep.* 7:6406. 10.1038/s41598-017-06402-w PMC5527005 28743969

[B126] Liedtke S. Enczmann J. Waclawczyk S. Wernet P. Kögler G. (2007). Oct4 and its pseudogenes confuse stem cell research. *Cell Stem Cell* 1 364–366. 10.1016/j.stem.2007.09.003 18371374

[B127] Liedtke S. Stephan M. Kögler G. (2008). Oct4 expression revisited: potential pitfalls for data misinterpretation in stem cell research. *Biol. Chem.* 389 845–850. 18627312 10.1515/BC.2008.098

[B128] Lilyanna S. Martinez E. C. Vu T. D. Ling L. H. Gan S. U. Tan A. L. (2013). Cord lining-mesenchymal stem cells graft supplemented with an omental flap induces myocardial revascularization and ameliorates cardiac dysfunction in a rat model of chronic ischemic heart failure. *Tissue Eng. Part A* 19 1303–1315. 10.1089/ten.tea.2012.0407 23448654 PMC3638562

[B129] Lim I. J. Phan T. T. (2014). Epithelial and mesenchymal stem cells from the umbilical cord lining membrane. *Cell Transplant.* 23 497–503. 10.3727/096368914x678346 24636188

[B130] Lindenmair A. Hatlapatka T. Kollwig G. Hennerbichler S. Gabriel C. Wolbank S. (2012). Mesenchymal stem or stromal cells from amnion and umbilical cord tissue and their potential for clinical applications. *Cells* 1 1061–1088. 10.3390/cells1041061 24710543 PMC3901122

[B131] Lisi A. Briganti E. Ledda M. Losi P. Grimaldi S. Marchese R. (2012). A combined synthetic-fibrin scaffold supports growth and cardiomyogenic commitment of human placental derived stem cells. *PLoS One* 7:e34284. 10.1371/journal.pone.0034284 22509287 PMC3317941

[B132] Loukogeorgakis S. P. Shangaris P. Bertin E. Franzin C. Piccoli M. Pozzobon M. (2019). In utero transplantation of expanded autologous amniotic fluid stem cells results in long-term hematopoietic engraftment. *Stem Cells* 37 1176–1188. 10.1002/stem.3039 31116895 PMC6773206

[B133] Lv F. J. Tuan R. S. Cheung K. M. Leung V. Y. (2014). Concise review: the surface markers and identity of human mesenchymal stem cells. *Stem Cells* 32 1408–1419. 10.1002/stem.1681 24578244

[B134] Ma J. Wu J. Han L. Jiang X. Yan L. Hao J. (2019). Comparative analysis of mesenchymal stem cells derived from amniotic membrane, umbilical cord, and chorionic plate under serum-free condition. *Stem Cell Res. Ther.* 10: 19. 10.1186/s13287-018-1104-x PMC6330472 30635045

[B135] Magatti M. Abumaree M. H. Silini A. R. Anzalone R. Saieva S. Russo E. (2016). The immunomodulatory features of mesenchymal stromal cells derived from Wharton’s Jelly, amniotic membrane and Chorionic Villi: *in vitro* and *in vivo* data. *Placenta* 2 91–128. 10.1201/b19620-6

[B136] Magatti M. Caruso M. De Munari S. Vertua E. De D. Manuelpillai U. (2015). Human amniotic membrane-derived mesenchymal and epithelial cells exert different effects on monocyte-derived dendritic cell differentiation and function. *Cell Transplant.* 24 1733–1752. 10.3727/096368914x684033 25259480

[B137] Magatti M. De Munari S. Vertua E. Gibelli L. Wengler G. S. Parolini O. (2008). Human amnion mesenchyme harbors cells with allogeneic T-cell suppression and stimulation capabilities. *Stem Cells* 26 182–192. 10.1634/stemcells.2007-0491 17901399

[B138] Maguire C. T. Demarest B. L. Hill J. T. Palmer J. D. Brothman A. R. Yost H. J. (2013). Genome-wide analysis reveals the unique stem cell identity of human amniocytes. *PLoS One* 8:e53372. 10.1371/journal.pone.0053372 23326421 PMC3542377

[B139] Manochantr S. Tantrawatpan C. Kheolamai P. Supokawej A. Issaragrisil S. (2010). Isolation, characterization and neural differentiation potential of amnion derived mesenchymal stem cells. *J. Med. Assoc. Thai.* 93(Suppl. 7) S183–S191. 21294413

[B140] Maraldi T. Bertoni L. Riccio M. Zavatti M. Carnevale G. Resca E. (2014). Human amniotic fluid stem cells: neural differentiation *in vitro* and *in vivo*. *Cell Tissue Res.* 357 1–13. 10.1007/978-3-319-23534-9_1 24788911

[B141] Mazza G. Roßmanith E. Lang-Olip I. Pfeiffer D. (2016). Marker profile for the evaluation of human umbilical artery smooth muscle cell quality obtained by different isolation and culture methods. *Cytotechnology* 68 701–711. 10.1007/s10616-014-9822-0 25535117 PMC4960121

[B142] Mennan C. Wright K. Bhattacharjee A. Balain B. Richardson J. Roberts S. (2013). Isolation and characterisation of mesenchymal stem cells from different regions of the human umbilical cord. *BioMed Res. Int.* 2013:916136. 10.1155/2013/916136 PMC3741948 23984420

[B143] Miki T. (2011). Amnion-derived stem cells: in quest of clinical applications. *Stem Cell Res. Ther.* 2:25. 10.1186/scrt66 21596003 PMC3152995

[B144] Miki T. Lehmann T. Cai H. Stolz D. B. Strom S. C. (2005). Stem cell characteristics of amniotic epithelial cells. *Stem Cells* 23 1549–1559. 10.1634/stemcells.2004-0357 16081662

[B145] Miki T. Marongiu F. Dorko K. Ellis E. C. S. Strom S. C. (2010). Isolation of amniotic epithelial stem cells. *Curr. Protoc. Stem Cell Biol.* 12 1E.3.1–1E.3.10. 10.1002/9780470151808.sc01e03s12 20049689

[B146] Miki T. Marongiu F. Ellis E. Strom C. S. (2007). Isolation of amniotic epithelial stem cells. *Curr. Protoc. Stem Cell Biol.* Chapter 1:Unit 1E.3. 10.1002/9780470151808.sc01e03s3 18785168

[B147] Miki T. Strom S. C. (2006). Amnion-derived pluripotent/multipotent stem cells. *Stem Cell Rev.* 2 133–142. 10.1007/s12015-006-0020-0 17237552

[B148] Miki T. Wong W. Zhou E. Gonzalez A. Garcia I. Grubbs B. H. (2016). Biological impact of xeno-free chemically defined cryopreservation medium on amniotic epithelial cells. *Stem Cell Res. Ther.* 7:8. 10.1186/s13287-015-0258-z PMC4711023 26758986

[B149] Moffett A. Loke C. (2006). Immunology of placentation in eutherian mammals. *Nat. Rev. Immunol.* 6 584–594. 10.1038/nri1897 16868549

[B150] Molbay M. Kipmen-Korgun D. Korkmaz G. Ozekinci M. Turkay Korgun E. (2018). Human trophoblast progenitor cells express and release angiogenic factors. *Int. J. Mol. Cell. Med.* 7 203–211. 31516879 10.22088/IJMCM.BUMS.7.4.203 PMC6709936

[B151] Montemurro T. Andriolo G. Montelatici E. Weissmann G. Crisan M. Colnaghi M. R. (2011). Differentiation and migration properties of human foetal umbilical cord perivascular cells: potential for lung repair. *J. Cell. Mol. Med.* 15 796–808. 10.1111/j.1582-4934.2010.01047.x 20219017 PMC3922668

[B152] Moodley Y. Atienza D. Manuelpillai U. Samuel C. S. Tchongue J. Ilancheran S. (2009). Human umbilical cord mesenchymal stem cells reduce fibrosis of bleomycin-induced lung injury. *Am. J. Pathol.* 175 303–313. 10.2353/ajpath.2009.080629 19497992 PMC2708816

[B153] Moorefield E. C. McKee E. E. Solchaga L. Orlando G. Yoo J. J. Walker S. (2011). Cloned, CD117 selected human amniotic fluid stem cells are capable of modulating the immune response. *PLoS One* 6:e26535. 10.1371/journal.pone.0026535 22046303 PMC3202543

[B154] Morley L. C. Shi J. Gaunt H. J. Hyman A. J. Webster P. J. Williams C. (2018). Piezo1 channels are mechanosensors in human fetoplacental endothelial cells. *Mol. Hum. Reprod.* 24 510–520. 10.1093/molehr/gay033 30085186 PMC6311101

[B155] Moschidou D. Drews K. Eddaoudi A. Adjaye J. De Coppi P. Guillot P. V. (2013a). Molecular signature of human amniotic fluid stem cells during fetal development. *Curr. Stem Cell Res. Ther.* 8 73–81. 10.2174/1574888x11308010009 23270629

[B156] Moschidou D. Mukherjee S. Blundell M. P. Jones G. N. Atala A. J. Thrasher A. J. (2013b). Human mid-trimester amniotic fluid stem cells cultured under embryonic stem cell conditions with valproic acid acquire pluripotent characteristics. *Stem Cells Dev.* 22 444–458. 10.1089/scd.2012.0267 23050522

[B157] Moser G. Drewlo S. Huppertz B. Armant D. R. (2018a). Trophoblast retrieval and isolation from the cervix: origins of cervical trophoblasts and their potential value for risk assessment of ongoing pregnancies. *Hum. Reprod. Update* 24 484–496. 10.1093/humupd/dmy008 29608700 PMC6016716

[B158] Moser G. Windsperger K. Pollheimer J. de Sousa Lopes S. C. Huppertz B. (2018b). Human trophoblast invasion: new and unexpected routes and functions. *Histochem. Cell Biol.* 150 361–370. 10.1007/s00418-018-1699-0 30046889 PMC6153604

[B159] Moser G. Gauster M. Orendi K. Glasner A. Theuerkauf R. Huppertz B. (2010). Endoglandular trophoblast, an alternative route of trophoblast invasion? Analysis with novel confrontation co-culture models. *Hum. Reprod.* 25 1127–1136. 10.1093/humrep/deq035 20176592

[B160] Moser G. Weiss G. Gauster M. Sundl M. Huppertz B. (2015). Evidence from the very beginning: endoglandular trophoblasts penetrate and replace uterine glands in situ and *in vitro*. *Hum. Reprod.* 30 2747–2757. 10.1093/humrep/dev266 26493408 PMC4719185

[B161] Moser G. Weiss G. Sundl M. Gauster M. Siwetz M. Lang-Olip I. (2017). Extravillous trophoblasts invade more than uterine arteries: evidence for the invasion of uterine veins. *Histochem. Cell Biol.* 147 353–366. 10.1007/s00418-016-1509-5 27774579 PMC5344955

[B162] Müller A. M. Hermanns M. I. Skrzynski C. Nesslinger M. Müller K. M. Kirkpatrick C. J. (2002). Expression of the endothelial markers PECAM-1, vWf, and CD34 *in Vivo* and *in Vitro*. *Exp. Mol. Pathol.* 72 221–229. 10.1006/exmp.2002.2424 12009786

[B163] Muñoz-Fernández R. de la Mata C. Prados A. Perea A. Ruiz-Magaña M. J. Llorca T. (2018). Human predecidual stromal cells have distinctive characteristics of pericytes: cell contractility, chemotactic activity, and expression of pericyte markers and angiogenic factors. *Placenta* 61 39–47. 10.1016/j.placenta.2017.11.010 29277270

[B164] Munoz-Fernandez R. Prados A. Leno-Duran E. Blazquez A. Garcia-Fernandez J. R. Ortiz-Ferron G. (2012). Human decidual stromal cells secrete C-X-C motif chemokine 13, express B cell-activating factor and rescue B lymphocytes from apoptosis: distinctive characteristics of follicular dendritic cells. *Hum. Reprod.* 27 2775–2784. 10.1093/humrep/des198 22718279

[B165] Murthi P. Hiden U. Rajaraman G. Liu H. Borg A. J. Coombes F. (2008). Novel homeobox genes are differentially expressed in placental microvascular endothelial cells compared with macrovascular cells. *Placenta* 29 624–630. 10.1016/j.placenta.2008.04.006 18514308

[B166] Murthi P. So M. Gude N. M. Doherty V. L. Brennecke S. P. Kalionis B. (2007). Homeobox genes are differentially expressed in macrovascular human umbilical vein endothelial cells and microvascular placental endothelial cells. *Placenta* 28 219–223. 10.1016/j.placenta.2006.02.012 16647116

[B167] Musiał-Wysocka A. Kot M. Sułkowski M. Badyra B. Majka M. (2019). Molecular and functional verification of Wharton’s Jelly mesenchymal stem cells (WJ-MSCs) pluripotency. *Int. J. Mol. Sci.* 20:1807. 10.3390/ijms20081807 31013696 PMC6515095

[B168] Nanaev A. K. Kohnen G. Milovanov A. P. Domogatsky S. P. Kaufmann P. (1997). Stromal differentiation and architecture of the human umbilical cord. *Placenta* 18 53–64. 10.1016/s0143-4004(97)90071-0 9032810

[B169] Niknejad H. Deihim T. Peirovi H. Abolghasemi H. (2013). Serum-free cryopreservation of human amniotic epithelial cells before and after isolation from their natural scaffold. *Cryobiology* 67 56–63. 10.1016/j.cryobiol.2013.05.001 23685252

[B170] Niknejad H. Khayat-Khoei M. Peirovi H. (2012). Inhibition of MMPs might increase anticancer properties of amniotic epithelial cells. *Med. Hypotheses* 78 690–691. 10.1016/j.mehy.2012.02.014 22401776

[B171] Niwa H. Miyazaki J. Smith A. G. (2000). Quantitative expression of Oct-3/4 defines differentiation, dedifferentiation or self-renewal of ES cells. *Nat. Genet.* 24 372–376. 10.1038/74199 10742100

[B172] Nursalim Y. N. S. Blenkiron C. Groom K. M. Chamley L. W. (2020). Growing human trophoblasts *in vitro*: a review of the media commonly used in trophoblast cell culture. *Reproduction* 160 R119–R128. 33112772 10.1530/REP-19-0605

[B173] Ochsenbein-Kölble N. Bilic G. Hall H. Huch R. Zimmermann R. (2003). Inducing proliferation of human amnion epithelial and mesenchymal cells for prospective engineering of membrane repair. *J. Perinat. Med.* 31 287–294. 12951883 10.1515/JPM.2003.040

[B174] Okae H. Toh H. Sato T. Hiura H. Takahashi S. Shirane K. (2018). Derivation of human trophoblast stem cells. *Cell Stem Cell* 22 50–63.e56. 29249463 10.1016/j.stem.2017.11.004

[B175] Olivares E. G. Montes M. J. Oliver C. Galindo J. A. Ruiz C. (1997). Cultured human decidual stromal cells express B7-1. (CD80). and B7-2. (CD86). and stimulate allogeneic T cells. *Biol. Reprod.* 57 609–615. 10.1095/biolreprod57.3.609 9282998

[B176] Oliveira M. S. Barreto-Filho J. B. (2015). Placental-derived stem cells: culture, differentiation and challenges. *World J. Stem Cells* 7 769–775. 10.4252/wjsc.v7.i4.769 26029347 PMC4444616

[B177] Oliver C. Cowdrey N. Abadia-Molina A. C. Olivares E. G. (1999). Antigen phenotype of cultured decidual stromal cells of human term decidua. *J. Reprod. Immunol.* 45 19–30. 10.1016/s0165-0378(99)00041-8 10660260

[B178] Palatnik A. Xin H. Su E. J. (2016). Dichotomous effects of aryl hydrocarbon receptor. (AHR). activation on human fetoplacental endothelial cell function. *Placenta* 44 61–68. 10.1016/j.placenta.2016.06.004 27452439 PMC4964606

[B179] Parolini O. Alviano F. Bagnara G. P. Bilic G. Buhring H. J. Evangelista M. (2008). Concise review: isolation and characterization of cells from human term placenta: outcome of the first international Workshop on Placenta Derived Stem Cells. *Stem Cells* 26 300–311. 10.1634/stemcells.2007-0594 17975221

[B180] Pavlicev M. Wagner G. P. Chavan A. R. Owens K. Maziarz J. Dunn-Fletcher C. (2017). Single-cell transcriptomics of the human placenta: inferring the cell communication network of the maternal-fetal interface. *Genome Res.* 27 349–361. 10.1101/gr.207597.116 28174237 PMC5340963

[B181] Pesce M. Wang X. Wolgemuth D. J. Schöler H. (1998). Differential expression of the Oct-4 transcription factor during mouse germ cell differentiation. *Mech. Dev.* 71 89–98. 10.1016/s0925-4773(98)00002-1 9507072

[B182] Phinney D. G. Galipeau J. (2019). Manufacturing mesenchymal stromal cells for clinical applications: a survey of Good Manufacturing Practices at U.S. academic centers. *Cytotherapy* 21 782–792. 10.1016/j.jcyt.2019.04.003 31182333

[B183] Piccoli M. Franzin C. Bertin E. Urbani L. Blaauw B. Repele A. (2012). Amniotic fluid stem cells restore the muscle cell niche in a HSA-Cre, Smn(F7/F7). mouse model. *Stem Cells* 30 1675–1684. 10.1002/stem.1134 22644669

[B184] Pipino C. Pierdomenico L. Di Tomo P. Di Giuseppe F. Cianci E. Morabito C. (2015). Molecular and phenotypic characterization of human amniotic fluid-derived cells. A morphological and proteomic approach. *Stem Cells Dev.* 24 1415–1428. 10.1089/scd.2014.0453 25608581

[B185] Pogozhykh O. Prokopyuk V. Figueiredo C. Pogozhykh D. (2018). Placenta and placental derivatives in regenerative therapies: experimental studies, history, and prospects. *Stem Cells Int.* 2018:4837930. 10.1155/2018/4837930 PMC5822788 29535770

[B186] Portmann-Lanz C. B. Schoeberlein A. Huber A. Sager R. Malek A. Surbek D. V. (2006). Placental mesenchymal stem cells as potential autologous graft for pre- and perinatal neuroregeneration. *Am. J. Obstet. Gynecol.* 194 664–673. 10.1016/j.ajog.2006.01.101 16522395

[B187] Pozzobon M. Piccoli M. Schiavo A. A. Atala A. De Coppi P. (2013). Isolation of c-Kit+ human amniotic fluid stem cells from second trimester. *Methods Mol. Biol.* 1035 191–198. 10.1007/978-1-62703-508-8_16 23959992

[B188] Pratama G. Vaghjiani V. Tee J. Y. Liu Y. H. Chan J. Tan C. (2011). Changes in culture expanded human amniotic epithelial cells: implications for potential therapeutic applications. *PLoS One* 6:e26136. 10.1371/journal.pone.0026136 22073147 PMC3206797

[B189] Prusa A. R. Hengstschlager M. (2002). Amniotic fluid cells and human stem cell research: a new connection. *Med. Sci. Monit.* 8 Ra253–Ra257. 12444390

[B190] Ramuta T. Kreft M. E. (2018). Human amniotic membrane and amniotic membrane-derived cells: How far are we from their use in regenerative and reconstructive urology? *Cell Transplant.* 27 77–92. 10.1177/0963689717725528 29562770 PMC6434475

[B191] Ramuta T. Starčič Erjavec M. Kreft M. E. (2020). Amniotic membrane preparation crucially affects its broad-spectrum activity against uropathogenic bacteria. *Front. Microbiol.* 11:469. 10.3389/fmicb.2020.00469 32265889 PMC7107013

[B192] Reza H. M. Ng B. Y. Phan T. T. Tan D. T. Beuerman R. W. Ang L. P. (2011). Characterization of a novel umbilical cord lining cell with CD227 positivity and unique pattern of P63 expression and function. *Stem Cell Rev. Rep.* 7 624–638. 10.1007/s12015-010-9214-6 21181306

[B193] Richards R. G. Brar A. K. Frank G. R. Hartman S. M. Jikihara H. (1995). Fibroblast cells from term human decidua closely resemble endometrial stromal cells: induction of prolactin and insulin-like growth factor binding protein-1 expression. *Biol. Reprod.* 52 609–615. 10.1095/biolreprod52.3.609 7756454

[B194] Ringden O. Baygan A. Remberger M. Gustafsson B. Winiarski J. Khoein B. (2018). Placenta-derived decidua stromal cells for treatment of severe acute graft-versus-host disease. *Stem Cells Transl. Med.* 7 325–331. 10.1002/sctm.17-0167 29533533 PMC5866941

[B195] Ringden O. Erkers T. Nava S. Uzunel M. Iwarsson E. Conrad R. (2013). Fetal membrane cells for treatment of steroid-refractory acute graft-versus-host disease. *Stem Cells* 31 592–601. 10.1002/stem.1314 23307526

[B196] Roffino S. Lamy E. Foucault-Bertaud A. Risso F. Reboul R. Tellier E. (2012). Premature birth is associated with not fully differentiated contractile smooth muscle cells in human umbilical artery. *Placenta* 33 511–517. 22495039 10.1016/j.placenta.2012.03.005

[B197] Rosner M. H. Vigano M. A. Ozato K. Timmons P. M. Poirier F. Rigby P. W. (1990). A POU-domain transcription factor in early stem cells and germ cells of the mammalian embryo. *Nature* 345 686–692. 10.1038/345686a0 1972777

[B198] Roy R. Kukucka M. Messroghli D. Kunkel D. Brodarac A. Klose K. (2015). Epithelial-to-mesenchymal transition enhances the cardioprotective capacity of human amniotic epithelial cells. *Cell Transplant.* 24 985–1002. 10.3727/096368913x675151 24256742

[B199] Ruetze M. Gallinat S. Lim I. J. Chow E. Phan T. T. Staeb F. (2008). Common features of umbilical cord epithelial cells and epidermal keratinocytes. *J. Dermatol. Sci.* 50 227–231. 10.1016/j.jdermsci.2007.12.006 18242061

[B200] Sakuragawa N. Kakinuma K. Kikuchi A. Okano H. Uchida S. Kamo I. (2004). Human amnion mesenchyme cells express phenotypes of neuroglial progenitor cells. *J. Neurosci. Res.* 78 208–214. 10.1002/jnr.20257 15378611

[B201] Salomon C. Ryan J. Sobrevia L. Kobayashi M. Ashman K. Rice G. E. (2013). Exosomal signaling during hypoxia mediates microvascular endothelial cell migration and vasculogenesis. *PLoS One* 8:e68451. 10.1371/journal.pone.0068451 23861904 PMC3704530

[B202] Sardesai V. S. Shafiee A. Fisk N. M. Pelekanos R. A. (2017). Avoidance of maternal cell contamination and overgrowth in isolating fetal chorionic villi mesenchymal stem cells from human term placenta. *Stem Cells Transl. Med.* 6 1070–1084. 10.1002/sctm.15-0327 28205414 PMC5442838

[B203] Sarugaser R. Hanoun L. Keating A. Stanford W. L. Davies J. E. (2009). Human mesenchymal stem cells self-renew and differentiate according to a deterministic hierarchy. *PLoS One* 4:e6498. 10.1371/journal.pone.0006498 19652709 PMC2714967

[B204] Schiavo A. A. Franzin C. Albiero M. Piccoli M. Spiro G. Bertin E. (2015). Endothelial properties of third-trimester amniotic fluid stem cells cultured in hypoxia. *Stem Cell Res. Ther.* 6:209. 10.1186/s13287-015-0204-0 PMC4628318 26519360

[B205] Schöler H. R. Dressler G. R. Balling R. Rohdewohld H. Gruss P. (1990). Oct-4: a germline-specific transcription factor mapping to the mouse t-complex. *EMBO J.* 9 2185–2195. 10.1002/j.1460-2075.1990.tb07388.x 2357966 PMC551941

[B206] Schütz M. Friedl P. (1996). Isolation and cultivation of endothelial cells derived from human placenta. *Eur. J. Cell Biol.* 71 395–401. 8980911

[B207] Sheridan M. A. Fernando R. C. Gardner L. Hollinshead M. S. Burton G. J. Moffett A. (2020). Establishment and differentiation of long-term trophoblast organoid cultures from the human placenta. *Nat. Protoc.* 15 3441–3463. 10.1038/s41596-020-0381-x 32908314

[B208] Shofuda T. Kanematsu D. Fukusumi H. Yamamoto A. Bamba Y. Yoshitatsu S. (2013). Human decidua-derived mesenchymal cells are a promising source for the generation and cell banking of human induced pluripotent stem cells. *Cell Med.* 4 125–147. 10.3727/215517912x658918 26858858 PMC4733846

[B209] Silini A. R. Cargnoni A. Magatti M. Pianta S. Parolini O. (2015). The long path of human placenta, and its derivatives, in regenerative medicine. *Front. Bioeng. Biotechnol.* 3:162. 10.3389/fbioe.2015.00162 26539433 PMC4609884

[B210] Silini A. R. Magatti M. Cargnoni A. Parolini O. (2017). Is immune modulation the mechanism underlying the beneficial effects of amniotic cells and their derivatives in regenerative medicine? *Cell Transplant.* 26 531–539. 10.3727/096368916x693699 27938500 PMC5661217

[B211] Silini A. R. Masserdotti A. Papait A. Parolini O. (2019). Shaping the future of perinatal cells: lessons from the past and interpretations of the present. *Front. Bioeng. Biotechnol.* 7:75 10.3389/fbioe.2019.00075I PMC6467938 31024907

[B212] Sölder E. Böckle B. C. Nguyen V. A. Fürhapter C. Obexer P. Erdel M. (2012). Isolation and characterization of CD133+CD34+VEGFR-2+CD45- fetal endothelial cells from human term placenta. *Microvasc. Res.* 84 65–73. 10.1016/j.mvr.2012.03.005 22480576

[B213] Soncin F. Khater M. To C. Pizzo D. Farah O. Wakeland A. (2018). Comparative analysis of mouse and human placentae across gestation reveals species-specific regulators of placental development. *Development* 145 dev156273. 10.1242/dev.156273 29361559 PMC5825847

[B214] Soncini M. Vertua E. Gibelli L. Zorzi F. Denegri M. Albertini A. (2007). Isolation and characterization of mesenchymal cells from human fetal membranes. *J. Tissue Eng. Regen. Med.* 1 296–305. 10.1002/term.40 18038420

[B215] Spitzhorn L. S. Rahman M. S. Schwindt L. Ho H. Wruck T. Bohndorf W. (2017). Isolation and molecular characterization of amniotic fluid-derived mesenchymal stem cells obtained from caesarean sections. *Stem Cells Int.* 2017:5932706. 10.1155/2017/5932706 PMC5684599 29225627

[B216] Stadler G. Hennerbichler S. Lindenmair A. Peterbauer A. Hofer K. van Griensven M. (2008). Phenotypic shift of human amniotic epithelial cells in culture is associated with reduced osteogenic differentiation *in vitro*. *Cytotherapy* 10 743–752. 10.1080/14653240802345804 18985480

[B217] Steigman S. A. Fauza D. O. (2007). Isolation of mesenchymal stem cells from amniotic fluid and placenta. *Curr. Protoc. Stem Cell Biol.* 1 1E.2.1–1E.2.12. 10.1002/9780470151808.sc01e02s1 18785167

[B218] Su E. J. Cheng Y. H. Chatterton R. T. Lin Z. H. Yin P. Reierstad S. (2007). Regulation of 17-beta hydroxysteroid dehydrogenase type 2 in human placental endothelial cells1. *Biol. Reprod.* 77 517–525. 10.1095/biolreprod.106.059451 17538076

[B219] Subramanian A. Fong C. Y. Biswas A. Bongso A. (2015). Comparative characterization of cells from the various compartments of the human umbilical cord shows that the Wharton’s Jelly compartment provides the best source of clinically utilizable mesenchymal stem cells. *PLoS One* 10:e0127992. 10.1371/journal.pone.0127992 26061052 PMC4464659

[B220] Sudo K. Kanno M. Miharada K. Ogawa S. Hiroyama T. Saijo K. (2007). Mesenchymal progenitors able to differentiate into osteogenic, chondrogenic, and/or adipogenic cells *in vitro* are present in most primary fibroblast-like cell populations. *Stem Cells* 25 1610–1617. 10.1634/stemcells.2006-0504 17395773

[B221] Tamagawa T. Oi S. Ishiwata I. Ishikawa H. Nakamura Y. (2007). Differentiation of mesenchymal cells derived from human amniotic membranes into hepatocyte-like cells *in vitro*. *Hum. Cell* 20 77–84. 10.1111/j.1749-0774.2007.00032.x 17645727

[B222] Terada S. Matsuura K. Enosawa S. Miki M. Hoshika A. Suzuki S. (2000). Inducing proliferation of human amniotic epithelial. (HAE). cells for cell therapy. *Cell Transplant.* 9 701–704. 10.1177/096368970000900518 11144969

[B223] Thormodsson F. R. Olafsson I. H. Vilhjalmsson D. T. (2018). Preparation and culturing of human primary vascular cells. *Methods Mol. Biol.* 1779 355–369. 10.1007/978-1-4939-7816-8_21 29886543

[B224] Troja W. Kil K. Klanke C. Jones H. N. (2014). Interaction between human placental microvascular endothelial cells and a model of human trophoblasts: effects on growth cycle and angiogenic profile. *Physiol. Rep.* 2:e00244. 10.1002/phy2.244 24760505 PMC4002231

[B225] Troyer D. L. Weiss M. L. (2008). Wharton’s jelly-derived cells are a primitive stromal cell population. *Stem Cells* 26 591–599. 10.1634/stemcells.2007-0439 18065397 PMC3311226

[B226] Tsai M. S. Lee J. L. Chang Y. J. Hwang S. M. (2004). Isolation of human multipotent mesenchymal stem cells from second-trimester amniotic fluid using a novel two-stage culture protocol. *Hum. Reprod.* 19 1450–1456. 10.1093/humrep/deh279 15105397

[B227] Tsang J. C. H. Vong J. S. L. Ji L. Poon L. C. Y. Jiang P. Lui K. O. (2017). Integrative single-cell and cell-free plasma RNA transcriptomics elucidates placental cellular dynamics. *Proc. Natl. Acad. Sci. U.S.A.* 114 E7786–E7795. 28830992 10.1073/pnas.1710470114 PMC5604038

[B228] Turco M. Y. Gardner L. Kay R. G. Hamilton R. S. Prater M. Hollinshead M. S. (2018). Trophoblast organoids as a model for maternal–fetal interactions during human placentation. *Nature* 564 263–267. 10.1038/s41586-018-0753-3 30487605 PMC7220805

[B229] Turco M. Y. Moffett A. (2019). Development of the human placenta. *Development* 146:dev163428. 10.1242/dev.163428 31776138

[B230] Underwood M. A. Gilbert W. M. Sherman M. P. (2005). Amniotic fluid: not just fetal urine anymore. *J. Perinatol.* 25 341–348. 10.1038/sj.jp.7211290 15861199

[B231] van Mourik J. A. Romani, de Wit T. Voorberg J. (2002). Biogenesis and exocytosis of Weibel-Palade bodies. *Histochem. Cell Biol.* 117 113–122. 10.1007/s00418-001-0368-9 11935287

[B232] Velicky P. Meinhardt G. Plessl K. Vondra S. Weiss T. Haslinger P. (2018). Genome amplification and cellular senescence are hallmarks of human placenta development. *PLoS Genet.* 14:e1007698. 10.1371/journal.pgen.1007698 30312291 PMC6200260

[B233] Vento-Tormo R. Efremova M. Botting R. A. Turco M. Y. Meyer K. B. Park J. E. (2018). Single-cell reconstruction of the early maternal-fetal interface in humans. *Nature* 563 347–353. 30429548 10.1038/s41586-018-0698-6 PMC7612850

[B234] Ventura Ferreira M. S. Bienert M. Muller K. Rath B. Goecke T. Oplander C. (2018). Comprehensive characterization of chorionic villi-derived mesenchymal stromal cells from human placenta. *Stem Cell Res. Ther.* 9:28. 10.1186/s13287-017-0757-1 PMC5800083 29402304

[B235] Verter F. Couto P. S. Bersenev A. (2018). A dozen years of clinical trials performing advanced cell therapy with perinatal cells. *Future Sci. OA* 4:Fso351. 10.4155/fsoa-2018-0085 30450234 PMC6234459

[B236] Vieira Paladino F. de Moraes Rodrigues J. da Silva A. Goldberg A. C. (2019). The immunomodulatory potential of Wharton’s Jelly Mesenchymal stem/stromal cells. *Stem Cells Int.* 2019:3548917. 10.1155/2019/3548917 PMC6594275 31281372

[B237] Vlahova F. Hawkins K. E. Ranzoni A. M. Hau K. L. Sagar R. David A. L. (2019). Human mid-trimester amniotic fluid. (stem). cells lack expression of the pluripotency marker OCT4A. *Sci. Rep.* 9:8126. 10.1038/s41598-019-44572-x PMC6544653 31148575

[B238] von Koskull H. (1984). Rapid identification of glial cells in human amniotic fluid with indirect immunofluorescence. *Acta Cytol.* 28 393–400. 6205529

[B239] von Koskull H. Aula P. Trejdosiewicz L. K. Virtanen I. (1984). Identification of cells from fetal bladder epithelium in human amniotic fluid. *Hum. Genet.* 65 262–267. 10.1007/bf00286514 6199284

[B240] Voyta J. C. Via D. P. Butterfield C. E. Zetter B. R. (1984). Identification and isolation of endothelial cells based on their increased uptake of acetylated-low density lipoprotein. *J. Cell Biol.* 99 2034–2040. 10.1083/jcb.99.6.2034 6501412 PMC2113570

[B241] Wang H. Yan X. Jiang Y. Wang Z. Li Y. Shao Q. (2018). The human umbilical cord stem cells improve the viability of OA degenerated chondrocytes. *Mol. Med. Rep.* 17 4474–4482. 29328479 10.3892/mmr.2018.8413 PMC5802223

[B242] Wang X. Athayde N. Trudinger B. (2004a). Microvascular endothelial cell activation is present in the umbilical placental microcirculation in fetal placental vascular disease. *Am. J. Obstet. Gynecol.* 190 596–601. 10.1016/j.ajog.2003.09.021 15041986

[B243] Wang H. S. Hung S. C. Peng S. T. Huang C. C. Wei H. M. Guo Y. J. (2004b). Mesenchymal stem cells in the Wharton’s jelly of the human umbilical cord. *Stem Cells* 22 1330–1337. 15579650 10.1634/stemcells.2004-0013

[B244] Wei J. P. Nawata M. Wakitani S. Kametani K. Ota M. Toda A. (2009). Human amniotic mesenchymal cells differentiate into chondrocytes. *Cloning Stem Cells* 11 19–26. 10.1089/clo.2008.0027 19226212

[B245] Whittle W. L. Gibb W. Challis J. R. G. (2000). The characterization of human amnion epithelial and mesenchymal cells: the cellular expression, activity and glucocorticoid regulation of prostaglandin output. *Placenta* 21 394–401. 10.1053/plac.1999.0482 10833375

[B246] Windsperger K. Dekan S. Pils S. Golletz C. Kunihs V. Fiala C. (2017). Extravillous trophoblast invasion of venous as well as lymphatic vessels is altered in idiopathic, recurrent, spontaneous abortions. *Hum. Reprod.* 32 1208–1217. 10.1093/humrep/dex058 28369440

[B247] Wolbank S. Peterbauer A. Fahrner M. Hennerbichler S. van Griensven M. Stadler G. (2007). Dose-dependent immunomodulatory effect of human stem cells from amniotic membrane: a comparison with human mesenchymal stem cells from adipose tissue. *Tissue Eng.* 13 1173–1183. 10.1089/ten.2006.0313 17518752

[B248] Wolbank S. van Griensven M. Grillari-Voglauer R. Peterbauer-Scherb A. (2010). Alternative sources of adult stem cells: human amniotic membrane. *Adv. Biochem. Eng. Biotechnol.* 123 1–27. 10.1007/10_2010_71 20237903

[B249] Wolfrum K. Wang Y. Prigione A. Sperling K. Lehrach H. Adjaye J. (2010). The LARGE principle of cellular reprogramming: lost, acquired and retained gene expression in foreskin and amniotic fluid-derived human iPS cells. *PLoS One* 5:e13703. 10.1371/journal.pone.0013703 21060825 PMC2966395

[B250] Wu M. Zhang R. Zou Q. Chen Y. Zhou M. Li X. (2018). Comparison of the biological characteristics of mesenchymal stem cells derived from the human placenta and umbilical cord. *Sci. Rep.* 8:5014. 10.1038/s41598-018-23396-1 PMC5864926 29568084

[B251] Xinaris C. Benedetti V. Novelli R. Abbate M. Rizzo P. Conti S. (2016). Functional human podocytes generated in organoids from amniotic fluid stem cells. *J. Am. Soc. Nephrol.* 27 1400–1411. 10.1681/asn.2015030316 26516208 PMC4849826

[B252] Yamahara K. Harada K. Ohshima M. Ishikane S. Ohnishi S. Tsuda H. (2014). Comparison of angiogenic, cytoprotective, and immunosuppressive properties of human amnion- and chorion-derived mesenchymal stem cells. *PLoS One* 9:e88319. 10.1371/journal.pone.0088319 24551087 PMC3925106

[B253] Yang F. Zheng Q. Jin L. (2019). Dynamic function and composition changes of immune cells during normal and pathological pregnancy at the maternal-fetal interface. *Front. Immunol.* 10:2317. 10.3389/fimmu.2019.02317 31681264 PMC6813251

[B254] Yen B. L. Huang H. I. Chien C. C. Jui H. Y. Ko B. S. Yao M. (2005). Isolation of multipotent cells from human term placenta. *Stem Cells* 23 3–9. 10.1634/stemcells.2004-0098 15625118

[B255] Yi X. Chen F. Liu F. Peng Q. Li Y. Li S. (2020). Comparative separation methods and biological characteristics of human placental and umbilical cord mesenchymal stem cells in serum-free culture conditions. *Stem Cell Res. Ther.* 11:183. 10.1186/s13287-020-01690-y PMC7238656 32430063

[B256] Zani A. Cananzi M. Lauriti G. Smith V. V. Bollini S. Ghionzoli M. (2014). Amniotic fluid stem cells improve survival and enhance repair of damaged intestine in necrotising enterocolitis via a COX-2 dependent mechanism. *Gut* 63 300–309. 10.1136/gutjnl-2012-303735 23525603

